# Evidence for human-centric in-vehicle lighting: Part 2—Modeling illumination based on color-opponents

**DOI:** 10.3389/fnins.2022.969125

**Published:** 2022-09-27

**Authors:** Christopher Weirich, Yandan Lin, Tran Quoc Khanh

**Affiliations:** ^1^Department of Illuminating Engineering and Light Sources, School of Information Science and Technology, Fudan University, Shanghai, China; ^2^Laboratory of Adaptive Lighting Systems and Visual Processing, Department of Electrical Engineering and Information Technology, Technical University of Darmstadt, Darmstadt, Germany

**Keywords:** in-vehicle illumination, psychological-light relation, light-scene preferences, dynamic-scenery, in-vehicle human factors

## Abstract

Illumination preference models are usually defined in a static scenery, rating common-colored objects by a single scale or semantic differentials. Recently, it was reported that two to three illumination characteristics are necessary to define a high correlation in a bright office-like environment. However, white-light illumination preferences for vehicle-occupants in a dynamic semi- to full automated modern driving context are missing. Here we conducted a global free access online survey using VR engines to create 360° sRGB static in-vehicle sceneries. A total of 164 participants from China and Europe answered three levels in our self-hosted questionnaire by using mobile access devices. First, the absolute perceptional difference should be defined by a variation of CCT for 3,000, 4,500, and 6,000 K or combinations, and light distribution, either in a spot- or spatial way. Second, psychological light attributes should be associated with the same illumination and scenery settings. Finally, we created four driving environments with varying external levels of interest and time of the day. We identified three key results: (1) Four illumination groups could be classified by applying nMDS. (2) Combinations of mixed CCTs and spatial light distributions outperformed compared single light settings (*p* < 0.05), suggesting that also during daylight conditions artificial light supplements are necessary. (3) By an image transformation in the IPT and CAM16 color appearance space, comparing external and in-vehicle scenery, individual illumination working areas for each driving scenery could be identified, especially in the dimension of chroma-, partially following the Hunt-Effect, and lightness contrast, which synchronizes the internal and external brightness level. We classified our results as a starting point, which we intend to prove in a follow-up-controlled laboratory study with real object arrangements. Also, by applying novel methods to display high fidelity 360° rendered images on mobile access devices, our approach can be used in the future interdisciplinary research since high computational mobile devices with advanced equipped sensory systems are the new standard of our daily life.

## Review of the mini-series

For the first time, led by artificial intelligence-based self-learning algorithms, the vehicle itself will be able to drive. Within this modern context of dynamic personal transportation, we started to investigate the role, purpose, and target of interior lighting in these modern vehicles.

In part one of our mini-series about human-centric in-vehicle lighting (Weirich et al., 2022), we investigated the application of ambient light in an indoor signaling context globally. We schematically designed luminaires in line shapes, positioned at state-of-the-art vehicle positions, and vary them by 10 different mono- and multichromatic colors to create specific visual stimuli for ambiance and messaging. We were able to identify, three color-preference groups with a polarizing, agreeing, or congruent expression. Next, only for the European participants, a strong hue dependency was observed for the mood of attention in the vehicle-signaling context but was missing for the Chinese group. Finally, within all groups, the door and the foot position were most favored for drivers and passengers.

In this present paper as the second part of our mini-series, we continue by separating light perception into three dimensions named brightness, color, and spatial distribution. At this time for passengers only, which are not driving, instead sitting in the second row and enjoying the trip at different times and place zones. We are going to introduce our study from a vision science point of view but with a focus on the visual pathway. First, we will introduce the opponent-color theory, as a model for color perception. Next, a short overview of recently developed indoor-lighting preference models and psychological illumination attributes are outlined. Finally, we combined all three fields in our study design and apply them to the modern vehicle context.

## Opponent-color theory and perceptual attributes

**Figure 1:**
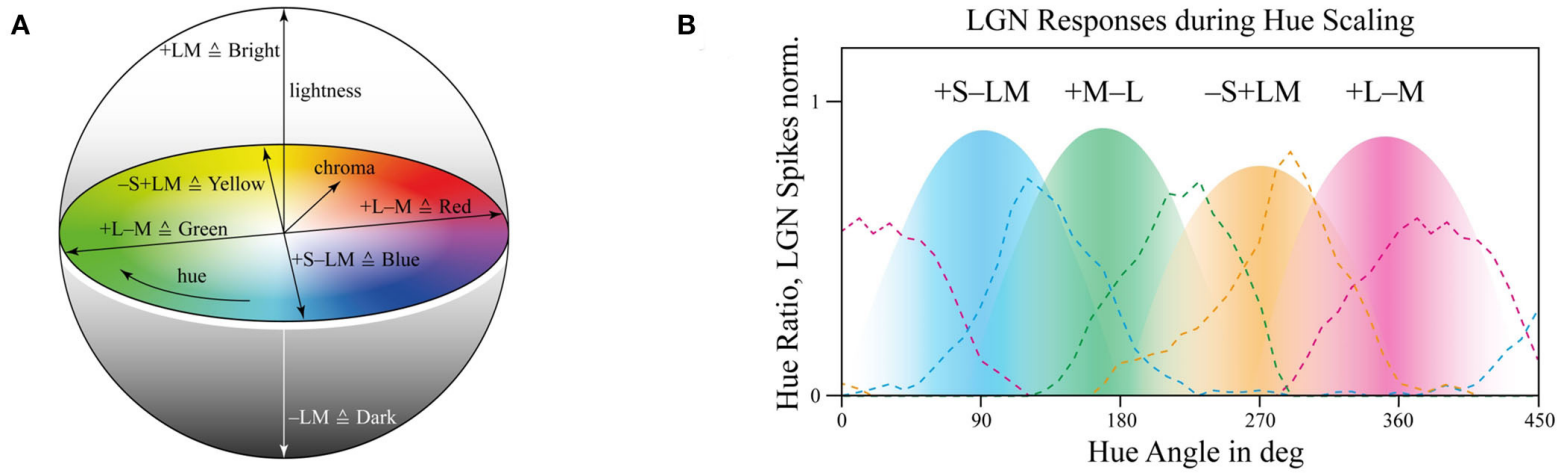
**(A)** Three-dimensional color sphere based on the opponent color theory with lightness axis for ± LM, red–green as ±L±M, and blue–yellow as ±S±LM including chroma and hue. **(B)** Hue ratios, in dotted lines, and retinal cellular opponent responses as surfaces, data from De Valois (2004).

Trichromats are able to perceive 100 hue shades per cone, leading to one million different perceived colors (Neitz et al., 2001) from 10^−6^ to 10^8^ or 10^9^ cd/m^2^ cd/m^2^ brightness levels, resulting in around 10^14^ magnitudes (Hood and Finkelstein, 1986). Pure and mixed colors are following in this perceptional process some regulations. Trichromats cannot perceive a reddish–green or a yellow–blue color, but they can see a yellow-reddish or a yellow-greenish color, which can be modeled in a 2D color circle (Newton, 1704) or with an extension by a third lightness dimension as shown in Figure 1A.

There are still accepted but also debated opinions about this color perception model since the underlying opponent-theory, a combination of polarizing and hyperpolarizing retinal cell responses, which will be outlined in the next paragraph, is challenged (Patterson et al., 2019). Anyway, here we still refer by applying the opponent-theory and focus on possible interpretations to use opponent signals from L-cone, M-cone, and S-cone, as illustrated in Figure 1B, and trying to transfer them in dimensions used to describe illumination perceptual quantities like lightness, hue or chroma settings.

Our three primary photoreceptors, L-cone, M-cone, and S-cone, at the retina, hyperpolarize to photon-stimuli in general (Schiller and Tehovnik, 2015). Hence, they are not able to distinguish changes in wavelengths by changes in intensity, which is called the principle of univariance, so a single dimension output only (Rushton, 1972). For that, intracellular connections compare the current activities of several receptors or receptive fields to extract visual information (Solomon and Lennie, 2007). Since the spectral absorption maximum between L- and M-cone is narrowly separated from each other, primary intensity can be triggered by them, resulting in the luminance axis for ± LM, which is also supported by the absence of S-cones in the fovea centralis (Ahnelt, 1998). But since there is still a ~30 nm peak gap between them, they are still able to process color information for the green as +M–L and red as a +L–M region. The S-cone signal absorption peak is wider separated by 90–120 nm between L- and S-cone. Hence, it is used to define a bluish color, +S–LM, or a more yellowish color, –S+LM (De Valois, 2004). This arrangement of perceiving only signal differences of receptive fields, no absolute values at all, can also lead to unequal color perceptions based on different bright backgrounds (Schrauf et al., 1997; Schiller and Tehovnik, 2015), which makes perceptual color modeling highly challenging.

Ebner's approach to finding this transformation between tristimulus activities and perceptual attributes resulted in a space called IPT (Ebner, 1998). His model should be extremely simple to implement to achieve linear hue lines and a neutral color response with accurate chroma representation to the Munsell data set. As a result, IPT performed better or at the same level as CIELAB or CIECAM97s (Ebner and Fairchild, 1998) especially for hue linearity to be able to calculate Euclidian distances for color difference calculations. The nonlinearity factor of 0.43 in IPT (Ebner, 1998) is very similar to gamma corrections defined in display systems between 0.45 and 0.55. Already in 1969, Marsden summarized exponential factors in the range of 0.15–0.59, depending on the size of the presented object, surrounded luminance, color of the presented stimulus, or level of eye adaptation (Marsden, 1969).

One model which includes higher non-linear luminance and chromatic adaption effects, like the Hunt–Effect (Hunt, 1977), which are intentionally excluded in IPT, was latest derived as CAM16 (Li et al., 2017), which solved computational failures during image processing of the previous CIECAM02 color-appearance model by CIE TC 8-01 (Moroney et al., 2002). Here we shortly summarize key points of it:

– Input values: *XYZ* tristimulus values.
– Output values: Correlates of lightness *J*, chroma *c*, hue composition *H*, hue angle *h*, colorfulness *M*, saturation *s*, and brightness *Q*.
– Uses a new chromatic adaptation transform as CAT16 and a new color appearance model named CAM16.
– To simplify and exclude computational errors, luminous and color adaptation are performed in the same space. Previously, CIECAM02 performed each transformation in its own space.
– CAM16 performed better in hue and chroma, lightness is similar to CIECAM02.
– Adding final transforms for uniform color space, CAM16-UCS, for Euclidian distances.

We selected IPT, based on the advantages that it originated from a newer dataset compared to CIELAB, improved hue linearity, especially in the bluish region (Moroney, 2003), and a simple formula approach. But since perceptual adaption effects are not included, we chose also CAM16 as a color appearance model, which gives us the possibility to compare the performance of both.

## Indoor illumination for user preference

Current investigated illumination models for general indoor lighting preferences are primarily derived by a triplet based on correlated color temperature (CCT), vertical illuminance (E_v_), and saturation enhancement (ΔC^*^; Trinh et al., 2019) or without intensity dependency in a linear relation between color gamut (CDI) and color fidelity (Q_a_; Huang et al., 2021). Further, combinations with non-visual parameters such as Circadian Stimulus (CS) were successfully established (Khanh et al., 2020). Also, preference models focusing on chroma found valid enhancements under dim light settings (Kawashima and Ohno, 2019). Chroma and color fidelity (Teunissen et al., 2017) or just gamut indices (Bao and Minchen, 2019) were combined in preference models and validated for different brightness areas. As identified, primary color metrics were applied for a high correlation between illumination settings and the observer's preferences. As a reference, one model was evaluated in a different context, applied in a museum environment, with a high correlation of 0.997 (Wang et al., 2020).

User preferences for illumination settings can be detailed in several psychological aspects as firstly identified by Flynn in the 1970's (Flynn et al., 1973). He varied brightness, luminaire position, and light distribution in an office-like environment to identify primary three different factors named as a general evaluative impression, perceptual clarity, and spaciousness. Similar attributes in the field of evaluative were named as relaxed or pleasant with perceptual clarity, spaciousness, and privacy (Durak et al., 2007). Also, psychological attributes in the same field of evaluative as attractive and perceptual quality with illumination were found (de Vries et al., 2018) or divided into coziness, liveliness, tenseness, and detachment, all out of the category of evaluative (Stokkermans et al., 2018).

So far, we were able to identify illumination preference models, which are primarily based on color metrics. These preferences can also be further divided into psychological aspects. Next, we will transfer these findings to the vehicle context.

## Transfer to human-centric in-vehicle lighting

In our investigated articles about color preferences and their psychological attributes for office lighting, there were primarily four blocking points identified preventing to project of these findings directly in the context of in-vehicle lighting: First, preference rating was performed on common-colored objects in a static office-like environment. Second, all ratings including semantic psychological differentials were performed in a static indoor environment without background changes. Third, the primary task for people in a vehicle is either driving or as a passenger to be transported from location A to B. Only in a second task, people will use in-vehicle lighting for their own doings like reading, listening to music, or relaxing by enjoying the outer scenery. For indoor lighting, light is directly connected to the primary task of people, which is to illuminate the scenery. Finally, vehicles are described more like smaller open boxes and dynamically change based on time and vehicle location, compared to the large wide office areas with fixed settings. In the following Table 1, these differences are summarized between indoor illumination and in-vehicle lighting (Wördenweber et al., 2007), based on extracts from indoor human-centric lighting recommendations (Khanh et al., 2022).

**Table 1:** Comparison of indoor lighting with in-vehicle lighting divided in luminaire recommendations and scene boundaries.

	**Indoor lighting**	**In-vehicle lighting**
**Luminaire recommendation:**		
Task-lighting, E_v_	500–625 l ×	1–100 l × depends on the function
Psychological glare, UGR	≤ 19	No–less glare, not specified
White light color preference	4,000 K < CCT < 5,800 K	Neutral white
CIE CRI *R*_a_	> 80	> 80
Spatial illumination	Indirect part > 60%	No shadow, homogeneous illumination
PWM Frequency	Min. 400 Hz, better > 1,000 Hz	488 Hz for RGB LEDs
**Scenery boundaries:**		
1. Evaluation	Rate common-colored objects.	Split: internal/external scene.
2. Location/surrounding	More static.	More dynamic.
3. People involved	Primary task: Connected to illumination, like reading. Secondary task: Not available.	Primary task: Driving/passenger transportation. Secondary task: Connected to illumination, like reading.
4. Box-setup	More closed box, large.	More open box, small.

Obviously, a simple transfer from indoor lighting to in-vehicle lighting is not possible. Hence, we focus on a new illumination preference modeling approach, away from colorimetric definitions like TM-30-20 (IES, 2020), which are defined and motivated by office-like sceneries by comparing light to a preset of defined color sample plates, as reviewed in Section Indoor illumination for user preference. Meaning, we will use tristimulus-based correlations of lightness, hue, and chroma, from the IPT or CAM16 color appearance space, compared in Section Opponent-color theory and perceptual attributes, to answer the following research question in the context of vehicle passengers and their psychological white-light associations, as reviewed in Section Indoor illumination for user preference:

q_1_: How many dimensions are necessary to describe in-vehicle lighting?
q_2_: In which ratio can psychological illumination-associated attributes be explained by these identified dimensions?
q_3_: How can in-vehicle lighting preferences be modeled based on tristimulus correlations in accordance with the change of the outer driving scenery?

## Materials and methods

To answer the research questions given in Section Transfer to human-centric in-vehicle lighting for the in-vehicle driving context, we conducted an online study, using VR-like pre-rendered images with dark-mode responsive web design techniques. The study was published on our self-hosted system and was globally free available. From the middle of April until the middle of June 2022, people were able to participate using their tablets, smartphone, or notebooks. Primary, we advertised our study using social media systems like Facebook, WhatsApp, and We-Chat. Participants could choose Chinese or English as their operating language. The study was divided into nine parts.

First, a short study-introduction movie was presented. Second, basic subject information was collected, which was similar to our first part (Weirich et al., 2022):

– Personal: Living region, gender, age class.
– Surrounding: Time of the day, weather conditions.
– Driving experience (without considering the COVID-19 pandemic): Time spent inside a vehicle during a normal week or if a subject drove a vehicle before.
– Social status: Acceptable price for buying a new vehicle and age of the subject's own vehicle.
– Visual performance: Contrast- and Ishihara test.

- Ishihara test: Prove that all participants have no color blindness.
- Contrast test: Prove that displayed text was readable, no external verification.

Bypassing all user information questions, the subject was forwarded to the device tracking page. Since we asked about white-light illumination settings, it is essential for the results of the study that each subject similarly views the lighting scenes. For that, we asked users to select their current device based on a drop-down list or type the brand and model properties manually if the device name was missing in our list. Next, a reminder was displayed to deactivate screen protection functions, like Apple's or Google's blueish eye protections, which are standard activated during evening time to block blueish light. Further, the user had to define their current screen brightness level by moving a slider to a similar position as the current system settings and finally a prompt appears not to change the current environment during the complete study. All this information can be used to measure photometric display properties like absolute screen brightness or color metrics. In addition, conclusions about the current surrounding of participants could be made, since the display brightness will change according to the current environmental brightness level, especially for mobile devices like smartphones or tablets.

In the fourth part, participants had to select absolute differences between 28 paired images, in an arbitrary combination of eight different in-vehicle illumination settings, as shown in Table 2. Here we varied CCT from cooler to warmer white 3,000, 4,500, and 6,000 K, similar to proposed for indoor illumination shown in Table 1, or combinations in two different spatial arrangements, called spot- or spherical illumination. In total, there were eight illumination settings, L1–L8, created. L1–L3 had a focused spotlight distribution, which changed from L5–L7 by adding more room-filling luminaire settings. L8 was defined as the baseline condition without in-vehicle lighting. The displayed modern in-vehicle scenery had in the first step no background scenery, shown as black windows, common-colored objects, like different fruits, a blueish shirt, a colorful magazine, and a neutral white interior with a blueish constant glass roof frame illumination for orientation.

**Table 2:** VR-prerendered images for arbitrary paired comparisons with illumination L1–L8.

(1)	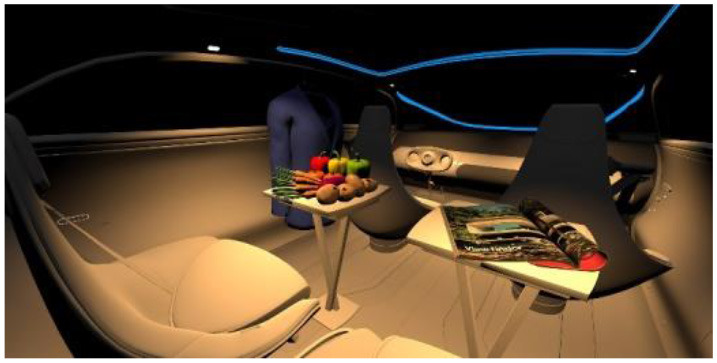	(2)	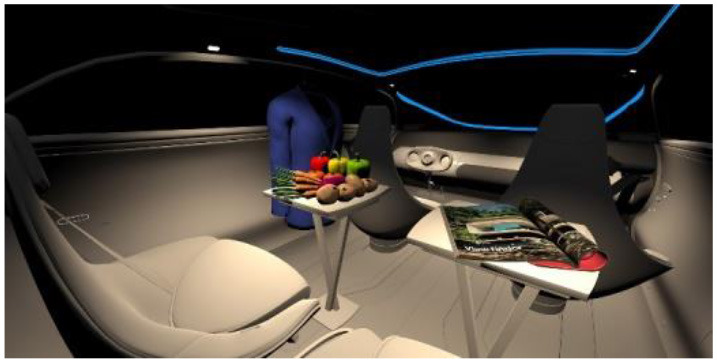
	Setting 1, L1: Spot light, 3,000 K.		Setting 2, L2: Spot light, 4,500 K.
(3)	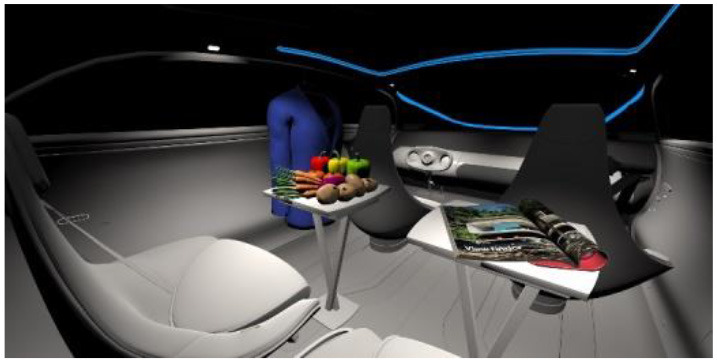	(4)	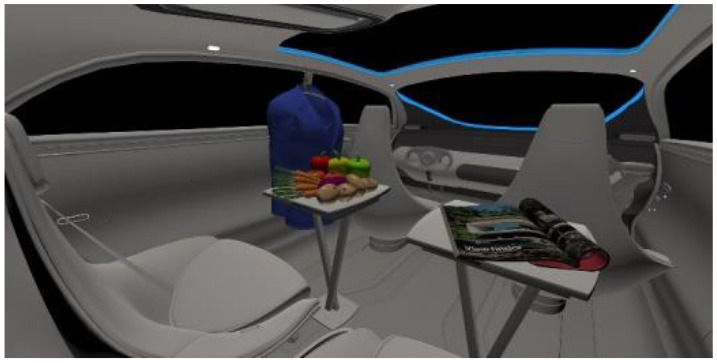
	Setting 3, L3: Spot light, 6,000 K.		Setting 4, L4: Spherical light, 6,000 K.
(5)	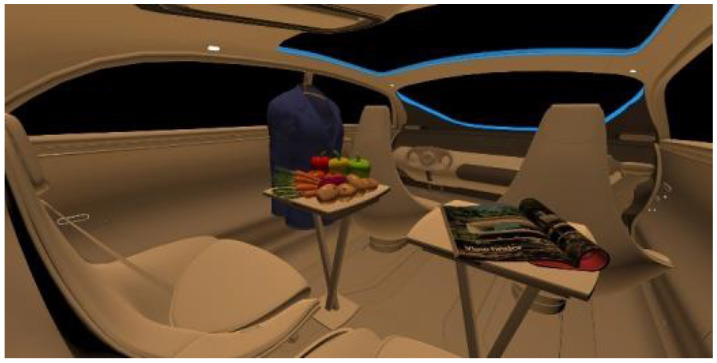	(6)	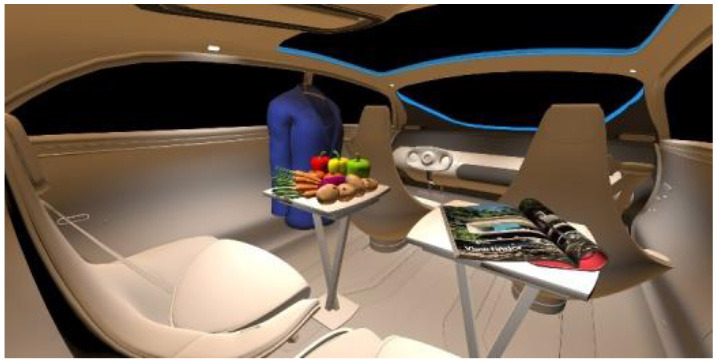
	Setting 5, L5: Spherical light, 3,000 K.		Setting 6, L6: Spherical light, 3,000 K + Spot light, 6,000 K.
(7)	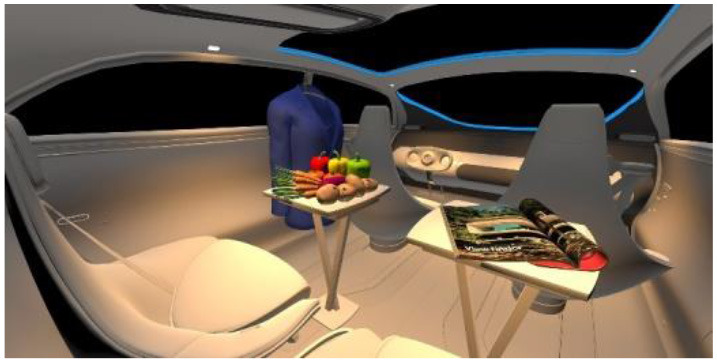	(8)	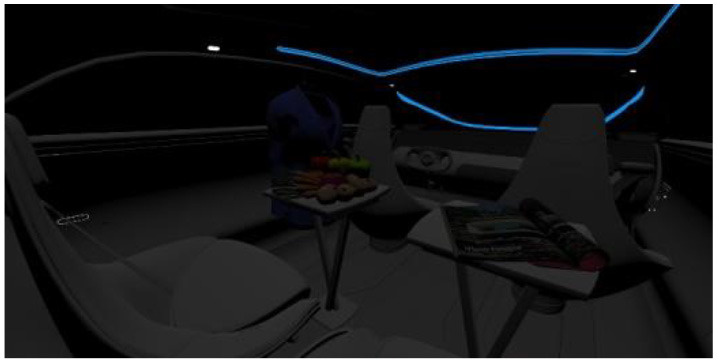
	Setting 7, L7: Spherical light, 6,000 K + Spot light, 3,000 K.		Setting 8, L8: No white light illumination.

To reset the visual system, we added after this comparison a short movie clip with white background. First, only a central black point was displayed. After several seconds, eight different graphical symbols were shown in a sequenced order, realized as the same movie clip for all participants. Each symbol was displayed for half a second, resulting in the left-right circulating pattern. To follow the symbols, eye saccades were stimulated, which were shown to be an efficient tool to reset and adapt the eye to the shown white background as new references (Paradiso et al., 2012). The complete reset clip took 16 s and a control question was added finally about the pattern of the last displayed symbol.

The sixth part of our online survey was focusing on associating psychological semantic differentials with the eight different illumination settings, as shown in Table 2. As reviewed in Section Indoor illumination for user preference, primary evaluative psychological effects were studied focusing on likeness, attractiveness, pleasantness, or coziness. On the other side, out of five investigated groups, Flynn et al. (1973) defined three which were correlated with luminaire settings named as evaluative impression, perceptual clarity, and spaciousness. Hence, we decided to investigate these three groups and selected one psychological attribute out of each group defined as interest, brightness, and spatiality. Further, we added additional three attributes matching more to the vehicle context named as modernity, value, and satisfaction. All six groups, including their three levels of semantic adjectives for differentiation, are added below, starting from agonist to antagonist order, which was presented as a drop-down menu during our study.

– Evaluative 1: Brightness

- Bright–Moderately Bright–Slightly Bright–Slightly Dark–Moderately Dark–Dark

– Evaluative 2: Spatial

- Large–Moderately Large–Slightly Large–Slightly Small–Moderately Small–Small

– Evaluative 3: Interest

- Interesting–Moderately Interesting–Slightly Interesting–Slightly Monotonous–Moderately Monotonous–Monotonous

– In-Vehicle 1: Modernity

- Modern–Moderately Modern–Slightly Modern–Slightly Old-Fashioned–Moderately Old-Fashioned–Old-Fashioned

– In-Vehicle 2: Value

- Valuable–Moderately Valuable–Slightly Valuable–Slightly Worthless–Moderately Worthless–Worthless

– In-Vehicle 3: Satisfaction

- Satisfied–Moderately Satisfied–Slightly Satisfied–Slightly Unsatisfied–Moderately Unsatisfied–Unsatisfied

The seventh part of our online survey had another round of resetting the vision system. A second movie clip was added based on different orders of shown symbols but following the same concept as written before.

In the eighth part, we created a higher immersive experience, illustrated in Table 3. By adding the external environment including the possibility to change the viewing perspective manually within the vehicle using pre-rendered 360° high-density images. Further, the user interaction level was also extended. We implemented the possibility that participants were able to select their preferred brightness setting by choosing five different levels. To illustrate these settings, Table 3 SPO.1–5 summarized the pre-rendered images as references for spot-light condition, L1, of 3,000 K and spatial lighting is shown in Table 3 SPA.1–5, L4, with 6,000 K. Here we varied intensities for spotlights between 5, 11, 25, 50, and 100% and spatial light between 10, 20, 30, 70, and 100%. The level of 100% was for both luminaires different to confirm that there will be no overexposure effect on the screen visible. We also skipped the single 4,500 K condition, L2, to just rate the outer boundaries of mixed CCTs and light distribution to minimize in a way the effort for study participants.

**Table 3:** Rating view of immersive 360° pre-rendered vehicle sceneries with spot light (SPO.1–5) and spherical lighting (SPA.1–5) including CCTs and applied brightness options in percentage values.

SPO.1	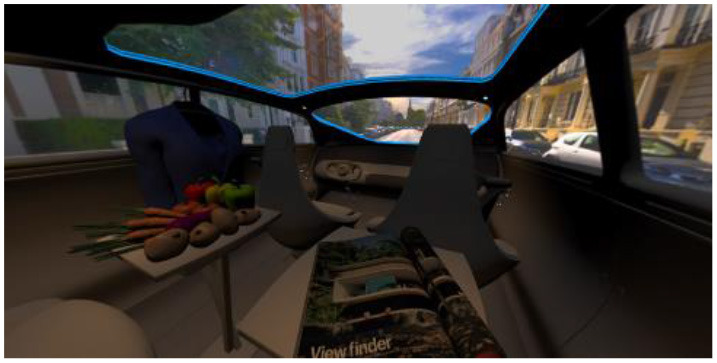	SPO.2	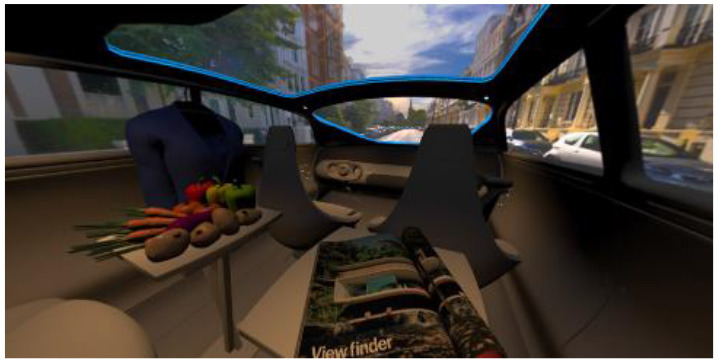
	City-Scene: Spot light, 3,000 K, 5%.		City-Scene: Spot light, 3,000 K, 11%.
SPO.3	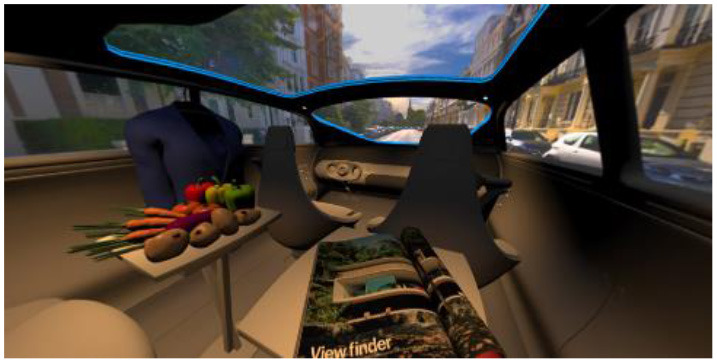	SPO.4	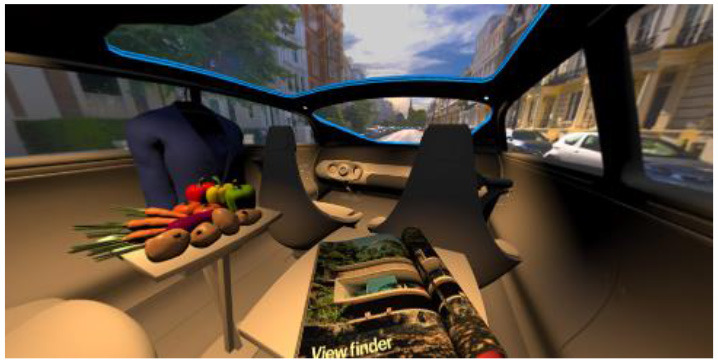
	City-Scene: Spot light, 3,000 K, 25%.		City-Scene: Spot light, 3,000 K, 50%.
SPO.5	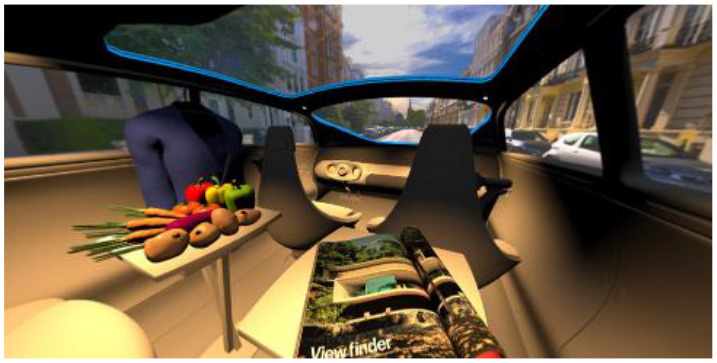	SPA.1	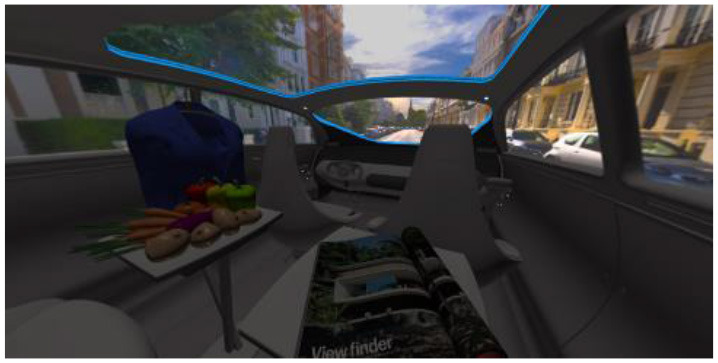
	City-Scene: Spot light, 3,000 K, 100%.		City-Scene: Spatial light, 6,000 K, 10%.
SPA.2	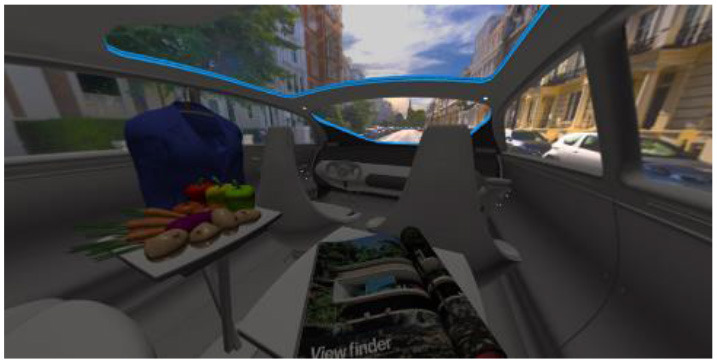	SPA.3	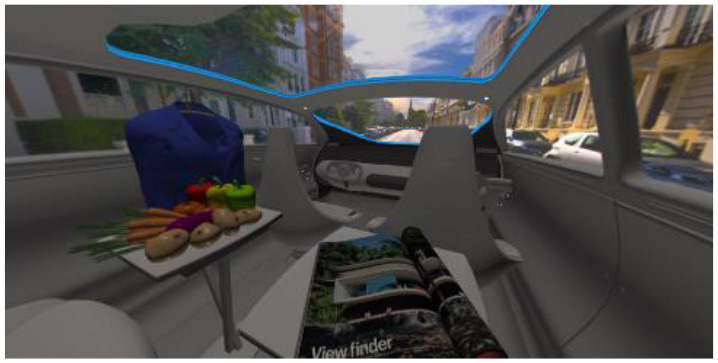
	City-Scene: Spatial light, 6,000 K, 20%.		City-Scene: Spatial light, 6,000 K, 30%.
SPA.4	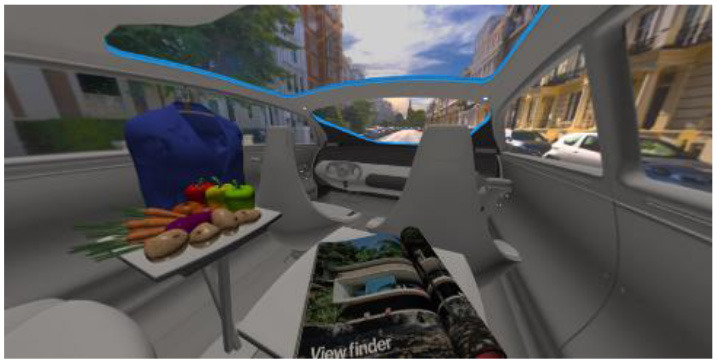	SPA.5	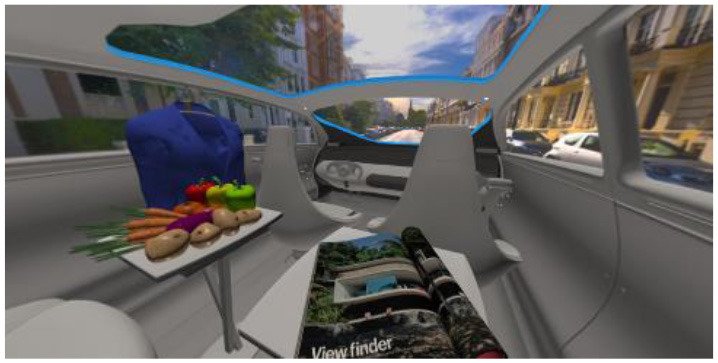
	City-Scene: Spatial light, 6,000 K, 70%.		City-Scene: Spatial light, 6,000 K, 100%.
SC.1	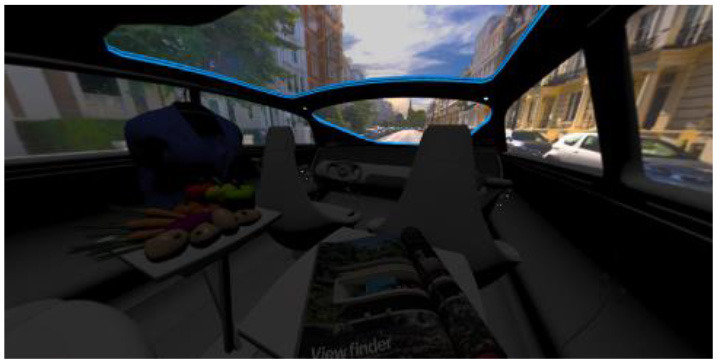	SC.2	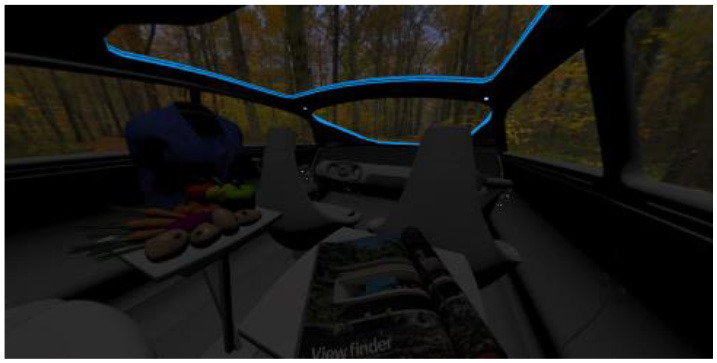
	Sun-City-Scene: Bright, interesting. No in-vehicle lighting, setting L8.		Forest-Scene: Dark, monotonous. No in-vehicle lighting, setting L8.
SC.3	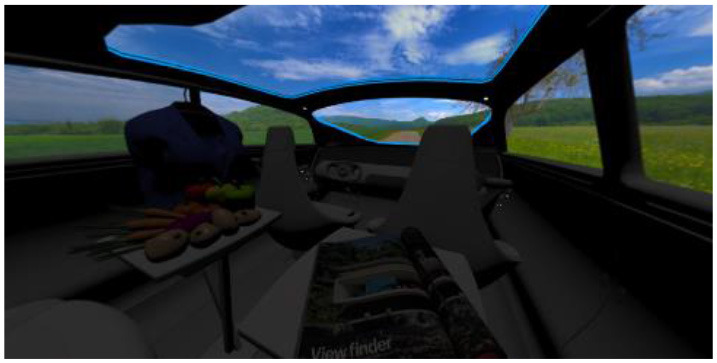	SC.4	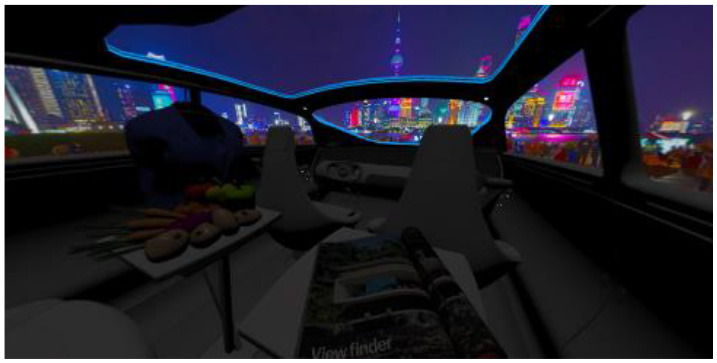
	Country-Scene: Bright, monotonous. No in-vehicle lighting, setting L8.		Night-Scene: Dark, interesting. No in-vehicle lighting, setting L8.

As reference for spot light, L1 and spherical light L4 settings are shown (SC.1–4). Overview of the four applied lighting sceneries, each without any white in-vehicle illumination and displayed during the survey in increased order.

In addition, we varied the external driving scene. Four different settings were chosen according to different light, color, and content settings, resulting in 124 pre-rendered images. The first scene showed a bright interesting sunny day driving through an inner-city area. The second one presented a darker monotonous forest scenery with very dim monotonous surrounding colors. The third scene presented a monotonous bright countryside scene with a blue sky and green grass views. Finally, we added a typical interesting colorful night scenery in Shanghai. As references, all four sceneries are shown in Table 3 SC.1–4 without interior lighting.

Illumination scenery rating was performed from a fixed defined view, which was set automatically after the preferred brightness level was activated. Here we asked about the user's preference on a 7-point scale named as excellent–very good–good–moderate–poor–bad–very bad, presented in a drop-down menu.

In the last part of our comprehensive survey, we asked subject to answer two qualitative optional questions.

i. Question 1, q_1_: Would you like to have interior lighting systems, which are changing according to the driving context? If yes, which lighting system do you want to have in your future vehicle?
ii. Question 2, q_2_: If you have some additional comments, please write down your opinions.

## Results

### Demographics

**Figure 2:**
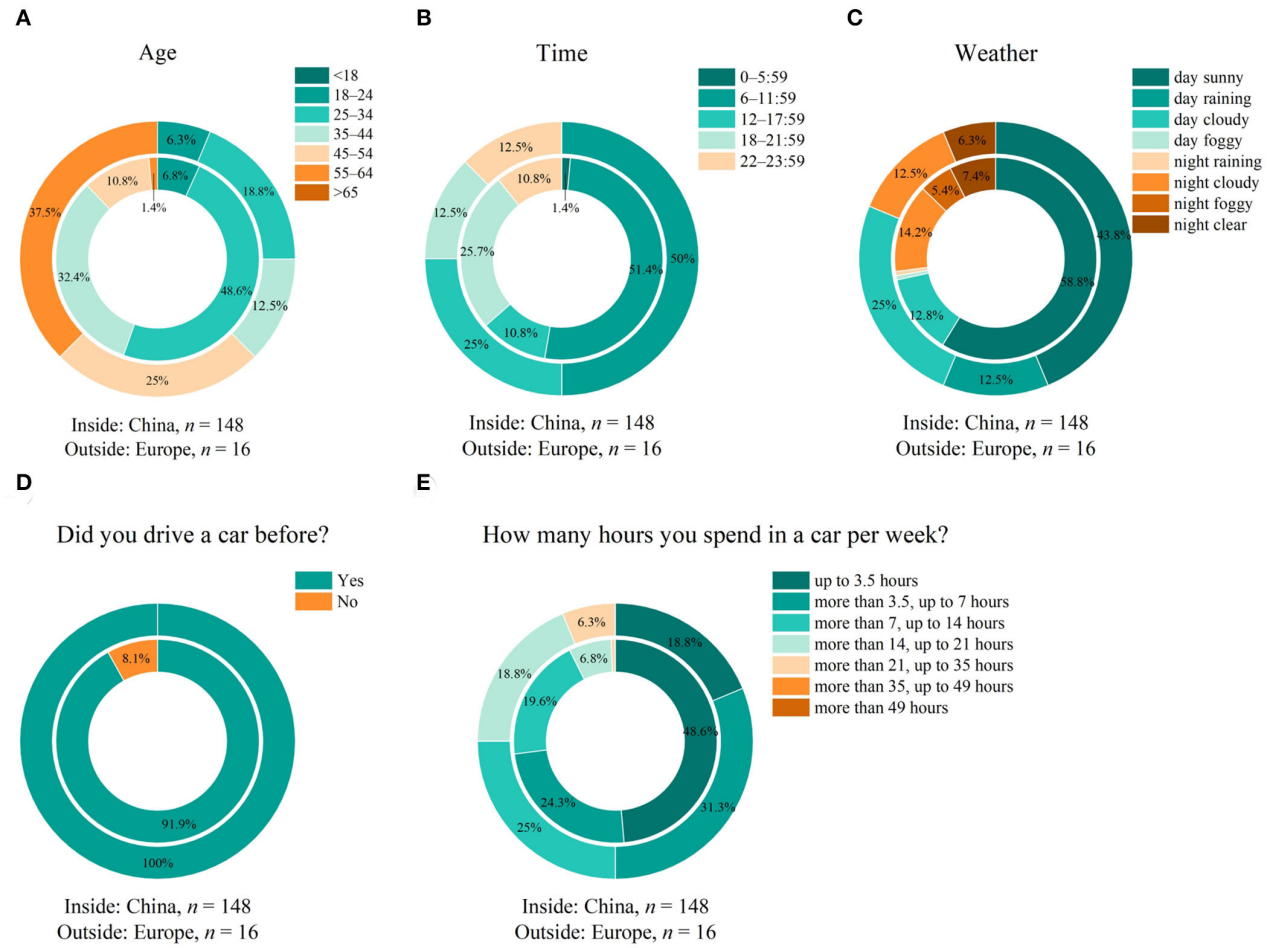
**(A)** Age, **(B)** time, and **(C)** weather settings of participants from China and Europe. **(D)** Driving habit of participants showing actual driving experience; **(E)** the amount of time spent in a vehicle per week.

We collected 164 answers from 120 male and 44 female participants. Since our target is a qualitative comparison between Chinese people from China with European people or ex-pats, which are living in China, we separated them accordingly to *China* and *Europe*. Out of the Europe group, 63% were directly located in Europe, and 37% were in China. The mean participated study time was t̄ = 23 min 48 s with a deviation of *s* = 14 min 31 s. Results about their participant age, time, and current weather conditions are shown in Figures 2A–C.

Since we performed our study anonymously, only age classes could be collected. Participants in the *Europe* group are on average 10 years older than people from *China*, as shown in Figure 2A. The average time of over 20 min is quite long compared to common online surveys and represents the comprehensive study scope within the nine survey parts. It might be also an indicator that participants really tried to answer all questions conscientiously as well. The participation attendance time was nearly balanced between both groups with 36% of participants from *China* participating from 6–0 PM, and in *Europe* around 25%. Comparing the day-night settings, for *China*, the separation was around 70:30 between day and night time and most of the people from *Europe* participated during the day as 80:20. The weather conditions were quite similar as well with the clearest or cloudy conditions during day and night time. Next, we identified the driving and vehicle habit of participants. Results are displayed in Figures 2D,E. It is shown that nearly all global participants drove a vehicle before by themselves. 70% of participants from group *China* drove up to one hour per week out of group *Europe*, there is 49% equivalent, showing a longer spend time per week in the vehicle in the *Europe* group.

### Illumination part I—Absolut differences

Statistical calculations within this study use the Wilcoxon signed rank test for dependent groups, since our data are ordinally scaled and there was no calculation comparing independent groups like Chinese and European participants. The null hypothesis *H*_0_ was formulated as an equal sample distribution, or concluded as an equal median, between two statistically compared groups. We calculated the asymptotic *p* and set the significance level α = 0.05. If the test result leads to α < *p, H*_0_ was rejected and the opposite hypothesis *H*_1_ would be valid, meaning the opposite of *H*_0_. The statistical effect size was calculated using Cohen's *r* (Cohen, 1988) including its semantic meaning for a weak, starting at *r* = 0.10, medium, starting at *r* = 0.25, or strong, starting at *r* = 0.40 (Fritz et al., 2012).

**Figure 3:**
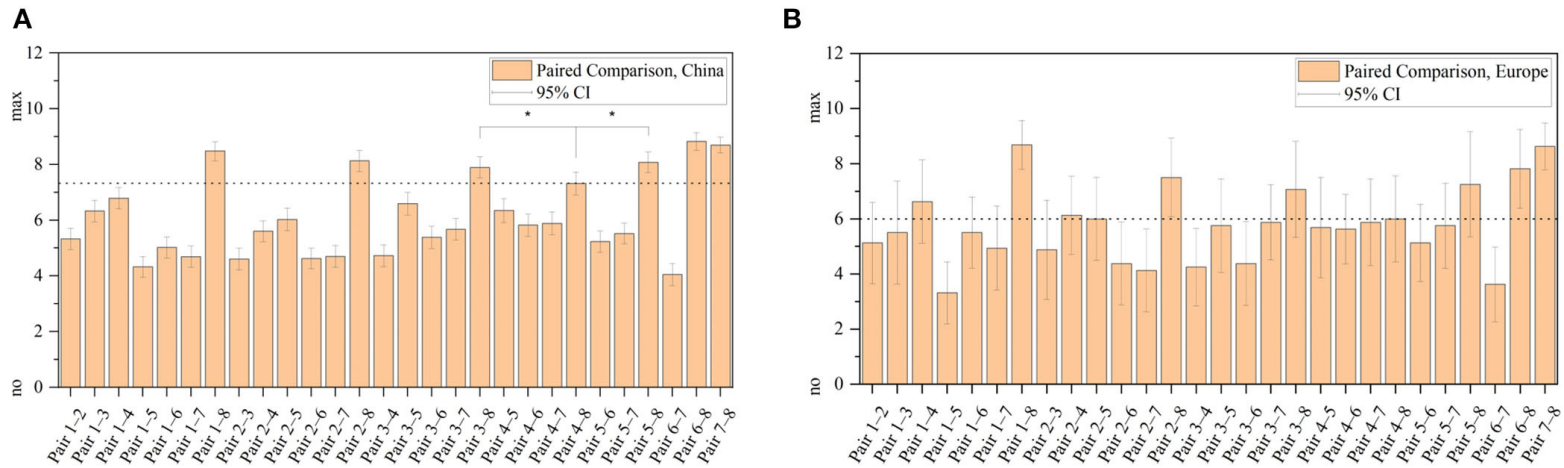
Twenty-eight paired comparisons from China **(A)** and Europe **(B)** rated from 0 as no difference to 10 as maximum deviation. Paired numbers can be referred out of Table 2. Ninety-five percent confidence interval shows an error of one step size in the Chinese group. Significantly closest relation to L8 is L4, spatial cool white, marked with * (*p* < 0.05) and as reference with a dashed line.

In this first survey part about white-light illumination preferences, we asked about absolute illumination differences, rated from 0 as no difference to 10 as maximum deviation, without any kind of participant's biasing. This means that we did not introduce or mentioned any kind of white-light scenery aspects, which should be compared. We just presented an arbitrary paired comparison out of eight in-vehicle lighting settings, L1–L8, as shown in Table 2, to find out the similarity level between L1 and L8. The results are presented in Figure 3.

As expected, the highest difference was observed comparing any illuminated setting with the no light baseline condition, L8. For *China*, the closest similarity to this index could be found at illumination index 4, representing 6,000 K spatial light distribution, L4. Calculated statistics *z* = −3.497, *p* = 4.69 × 10^−4^, and Cohen's coefficient *r* = 0.287, resulting in medium effect size between pairs L4–L8 and L3–L8.

**Figure 4:**
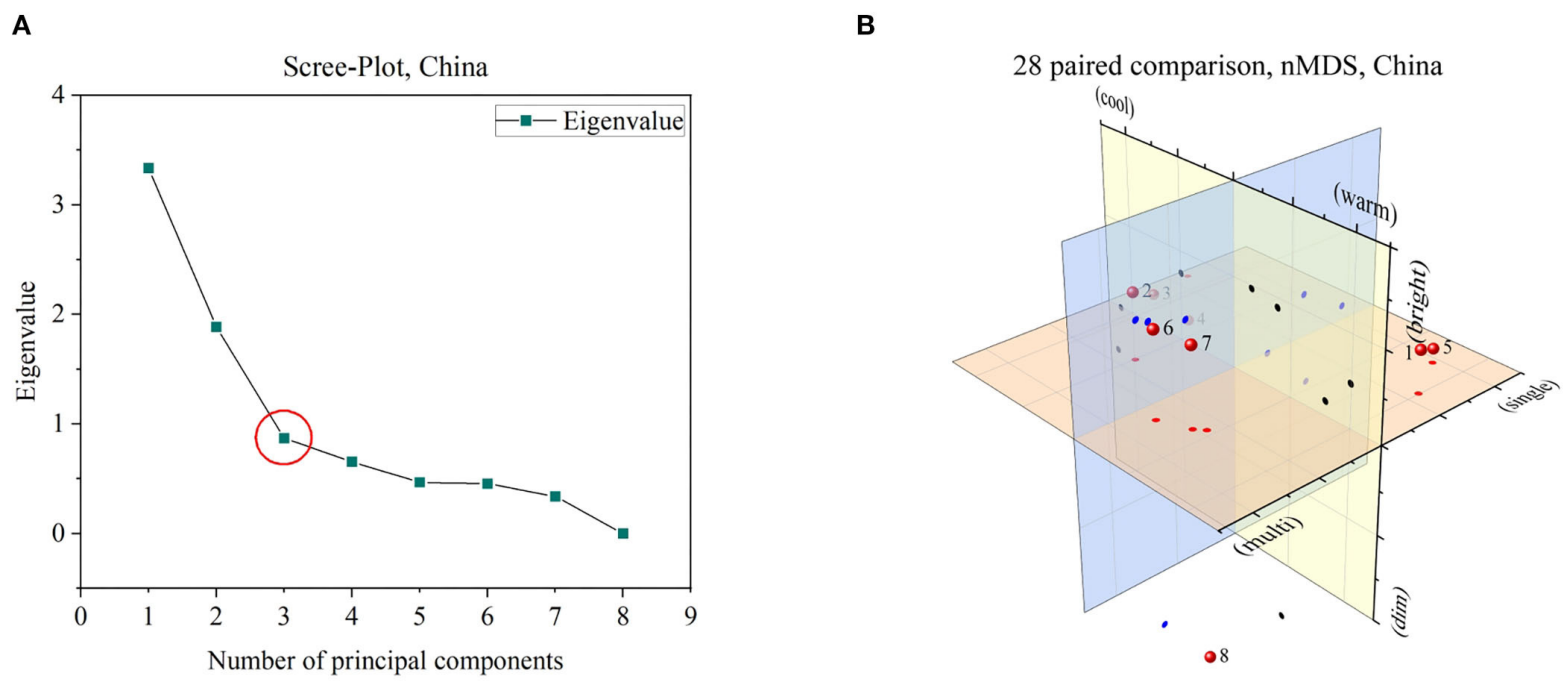
**(A)** By setting three dimensions, resulted stress value of nMDS equals zero, which can be confirmed by a Scree-plot as well, marked with a red circle. **(B)** Three identified dimensions roughly labeled by illumination characteristics. Numbers 1–8 are representing lighting sceneries L1–L8. Plane projects are added: Red circles for the red plane, blue circles for the blue plane, and black circles for the yellow plane.

With these results, we performed a non-metric multidimensional scaling (nMDS) to identify how many different dimensions are necessary to describe the similarity of the L1–L8 settings. For a comparison judgment, only the Chinese participants were investigated based on the smaller confidence intervals, shown in Figure 3A. Since our data scale is ordinal, only rank comparisons could be used. Principal component analysis is using Euclidian distances, which require an absolute zero. So we used the Bray–Curtis dissimilarity matrix, which actually does not calculate distances but dissimilarities because of using rank comparisons leading to robust distance results (Faith et al., 1987). By applying three dimensions, the resulting stress value is zero, meaning that three dimensions are necessary to describe the paired-comparison dataset. For reference only, a Scree-Diagram is added to get a more comprehensive overview of the number of necessary dimensions, to extend the interpretation of the stress value. By using three principal components around 76% of all differences could be explained. Figure 4 shows the Scree diagram and the resulting non-metric multidimensional scaling, plotted in three dimensions.

Out of the nMDS analysis, the following dimensions were preliminarily named based on identified groups and their optical properties.

– Warm-Cool-Dimension, marked with red circles: Separated luminaire groups had indices L1, L5, L6, L7, and (L8) characterized by primary warmer white or mixed CCT conditions vs. L2, L3, and L4 with primary cooler or neutral white settings.
– Single-Multi-Dimension, marked with blue circles: Separated luminaire groups had indices L1, L3, L4, and L5 characterized by primary single luminaire or single CCT conditions versus L2, L6, L7, and (L8) with primary multiple CCT or luminaire settings.
– Bright-Dim-Dimension, marked with black circles: Separated luminaire groups had indices L4, L8 characterized by primary darker conditions versus L1, L2, L3, L5, L6, and L7 with primary brighter luminaire settings.

### Illumination part II—Psychological attributes

To investigate previously roughly defined dimensions, we added six psychological attributes presented in a semantic differential way. Three of these named brightness, spatial, and interest were extracted out of the previous overview, and compared in Section Indoor illumination for user preference. The last three attributes were set as modernity, value, and satisfaction, to define a closer connection to the driving or the in-vehicle context.

**Figure 5:**
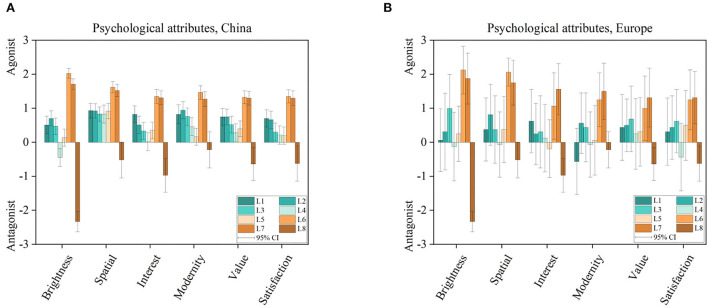
Six psychological attributes rated on a semantic scale from 3 as highly supporting to −3 as highly contradiction associated with eight illumination settings L1–L8, compare Table 2, evaluated in the Chinese **(A)** and European **(B)** group.

As shown in Figure 5, from 3 as highly supporting to−3 as high contradiction, within all six categories mixed CCT and spatial luminaire settings, L6 and L7, so a mixture of spatial- and spot light with warmer and cooler CCT, performed extraordinarily with a very high level of agreeing in all categories. One reason for this might be that both luminaires combine advantages of (a) a focused bright illumination to closer objects which are interesting, like the shown fruits or magazines and (b) also consider the complete vehicle interior as a room filling setting as well. Combining these two aspects might be led to a new level of vehicle perception. For the category of brightness, L4 as spatial 6,000 K, and L8, as the no light condition, were darker rated, matching our nMDS analysis before. A spatial increment in room perception was similar within different CCTs, spatial- or spot-light systems rated as slightly large. But also, for the no-light baseline condition in which just the blue roof frame illumination was visible, the room had still an impression of being only slightly small. For the attribute of interest, warmer white light had a higher level of interest and only L8 resulted in a monotonous impression. For the vehicle attributes, single CCTs were able to create only an impression of slightly modern, which was also close to the interpretation of the baseline condition as well. A similar relation was associated with the psychological attribute of value and satisfaction. In this group, warmer CCTs performed better compared to cooler ones.

### Illumination part III—Modeling preference with opponent colors

In this part, we added four external driving sceneries as introduced in Table 3. Also, participants were able to select their preferred illumination brightness out of five different levels. To bring a higher level of experience, maybe the highest possibility within this open-access study design, we pre-rendered the seven mentioned luminaire conditions L1 + L3-L8 since L2 was decided to skip to save answering effort, with five different brightness and four different external sceneries, created a dataset of 124 images rendered in a 360° style in 1,024 × 512-pixel resolution. Normally, these renderings need a high computational environment with several dedicated graphics processing units. To overcome this challenge, we found a possibility to accelerate the rendering progress by using the web graphics library (WebGL) for web browsers. For the scenery design, we used professional rendering software named 3ds Max 2022^®^. The rendering itself was then performed using WebGL techniques, resulting in a dramatically decreased rendering time to nearly real-time.

**Figure 6:**
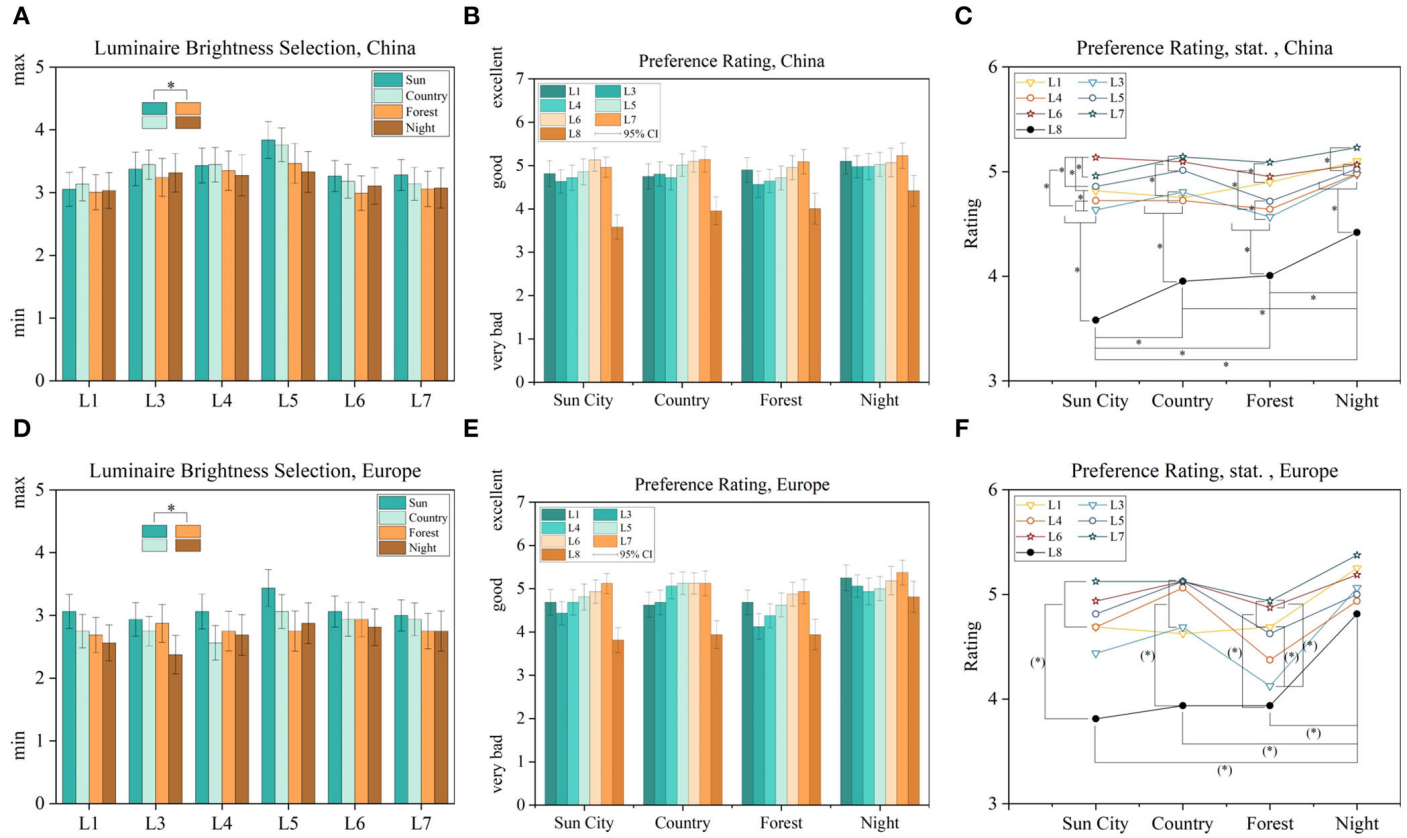
Luminaire brightness selections between four different external sceneries **(A,D)**, its preference rating **(B,E)** and statistics of it **(C,F)** separated between Europe and China. Significances for *p* < 0.05 are marked with ^*^ or *p* < 0.08 as (^*^).

In Figure 6 the results are displayed of the selected brightness values and their preference over seven light settings and four different driving sceneries. Primary, we will evaluate results from the bigger sampled group out of China. Starting with a comparison of the selected brightness values, a high correlation between brighter and darker external sceneries was found, as shown in Figures 6A,D. The ratings of sun city with countryside were combined and compared with combinations of forest and night scenery to perform a non-parametrical combined sample test for dependent groups, here the Wilcoxon signed-rank test. We calculated *z* = 2.417 and *p* = 1.566 × 10^−3^. The effect size was calculated with Cohen's coefficient *r* = 0.198 (Cohen, 1988), resulting in weak effect size (Fritz et al., 2012). For the preference rating, presented in Figures 6B,C, the highest effect was found between L6 and L8, with no in-vehicle light condition, in the sun city scenery, calculated as *z* = 8.352, *p* = 0.000 and *r* = 0.687, resulting in a strong effect size for the Chinese group. Further, for the countryside and forest scenery, luminaire groups were identified with similar performance (*p* < 0.05), meaning that within the good ranking for lighting settings L1–L7, warmer white colors are suggested to be preferred like L1, L5 with the mixed CCT favorites of L6 and L7. Only during the night condition, L8 was able to achieve a moderate–good acceptance level. Further points are analyzed in the next paragraphs. Complete statistical tables are added in the Supplementary Tables 1–10.

**Figure 7:**
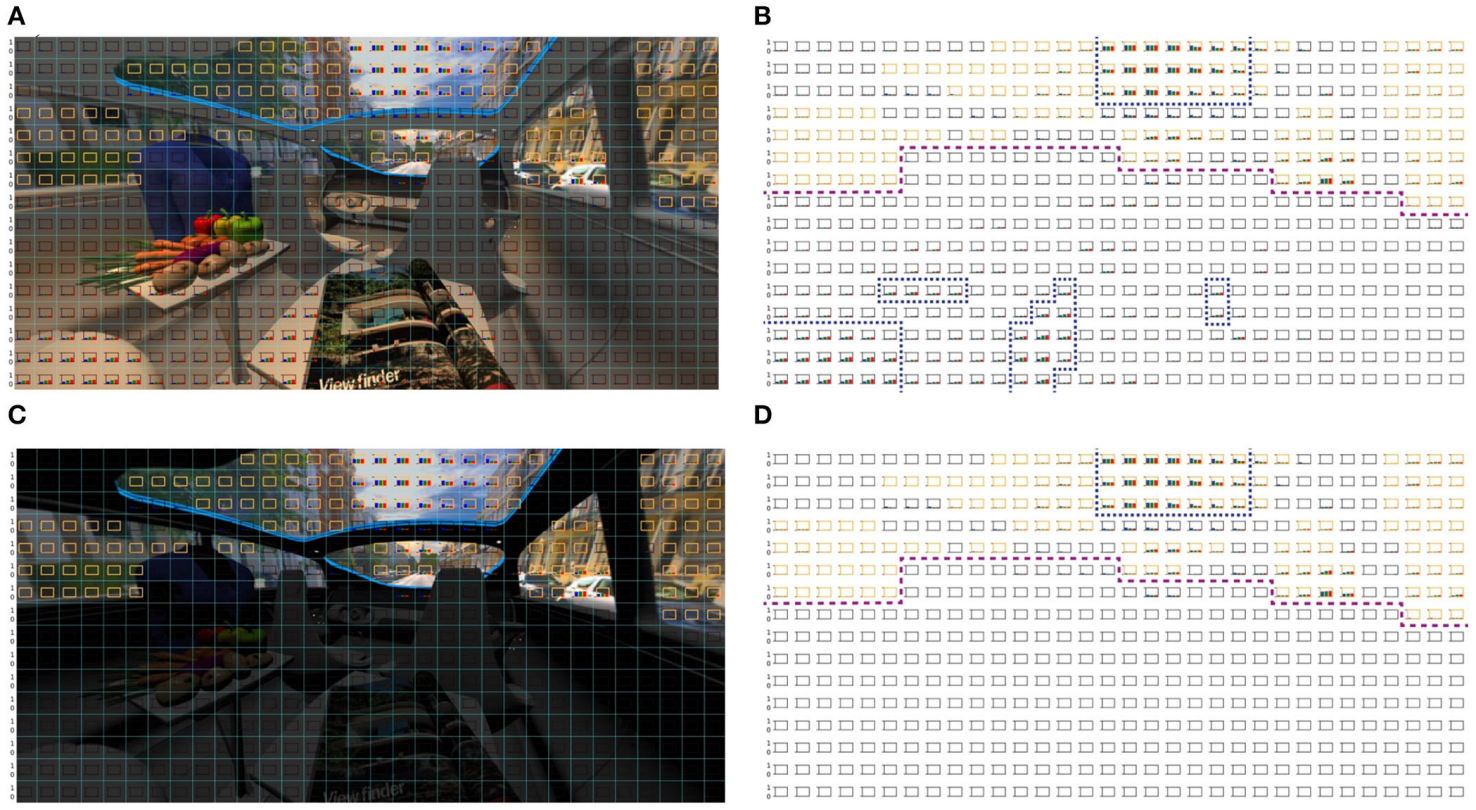
**(B,D)** Primary boundary line, dashed purple, between exterior and interior. Highest LMS activation for exterior and interior marked in dashed blue. **(A,B)** Mixed CCT condition, L7, rated as good illumination in sun-city scenery and its LMS activation profile. **(C,D)** No in-vehicle lighting, which was worse rated. Primary LMS activation area is marked in blue for exterior and interior. Orange pixel blocks represent the outside scenery.

So far, we were able to primarily identify a high correlation between in-vehicle brightness and outside brightness levels, Figures 6A,D. To investigate this further, we performed a transformation between the displayed sRGB images to IPT and CAM16 color appearance space. The advantages of these perceptional color spaces are explained in Section Indoor illumination for user preference. IPT is based on tristimulus values out of the LMS space. To investigate the suitability of the LMS space, we transformed the worst and best rated VR 360° scenery from its rating perspective, an 86° field of view, both to LMS space. For that, we divided the image with a resolution of 1,024 × 512 pixels into 32 × 32 pixel-blocks per square resulting in 512 fields. Out of each field, we calculated mean LMS values, which are displayed in Figure 7.

It is shown that there is an in-vehicle LMS activation in a good rated lighting condition, like L7, shown in blue dashed lines in Figure 7B. Within the L8 condition, Figure 7D, the activation is primarily achieved based on external sceneries only, since inside the vehicle light is totally missing. We marked areas describing the external scenery, from its rating perspective, in orange and kept other squared pixels blank leading to a ratio of 25% external illumination and 75% in-vehicle illumination. Further, purple dashed lines divide the external and the internal scenery roughly for orientation.

Next, we transformed the images in IPT-space and calculated lightness, chroma, and hue according to equation published by Ebner (1998). But since our vision system is triggered by contrasts instead of absolute values, we calculated contrast values of previous dimensions according to Equation 1 where *sc* stands for external scenery, which are the orange marked areas in Figure 7, and *il* for in-vehicle scenery, which describes all other pixel-boxes.


(1) $$\begin{array}{l} {Contrast\;}_{J,c,h}=\;\frac{abs\left(sc\right)-abs\left(il\right)}{abs\left(sc\right)+abs\left(il\right)}•100\;\left[\%\right] \end{array}$$

**Figure 8:**
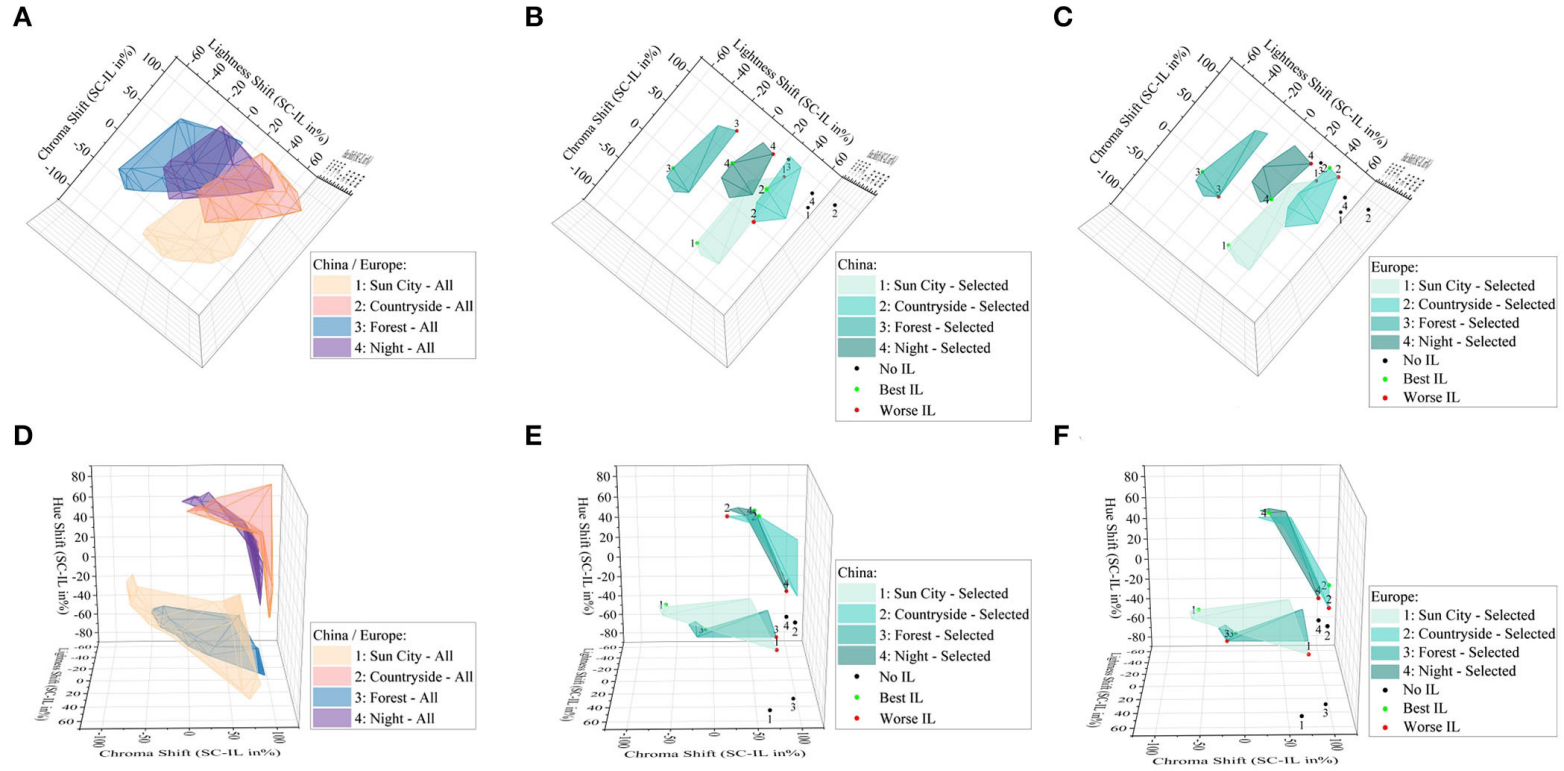
**(A–C)** 3D plots of lightness shift Δ*J*, **(A–C)** chroma shift Δ*c* and **(D–F)** hue shift Δ*h*. **(A–F)** Plots are rotated only to show single dimensions. Maximum possible lightness and chroma shift within all displayed luminaire and brightness settings **(A)** and hue ranges **(D)**. Selected preferences **(B,C)** according to best and worst ratings as displayed in Figures 6B,C. Hue differences between best and worst **(E,F)**. All separated between the Europe and China group.

Based on this equation, we were able to calculate contrast spaces of lightness, chroma and hue for all lighting settings including all brightness, CCT, and luminaire spatial distributions or three basic illumination categories. Following, we were able to identify first, all possible lightness, chroma, and hue settings, shown in Figures 8A,D. Further, we were able to shrink this area to preference levels, following ratings from Figures 6C,F, which are displayed in Figures 8B,C in the chroma and lightness space and in Figures 8E,F for the hue space. This means that we are able to define perceptional working areas based on each external scenery. Here we resorted to the sceneries according to external brightness settings. Meaning from external brighter to darker settings.

**Figure 9:**
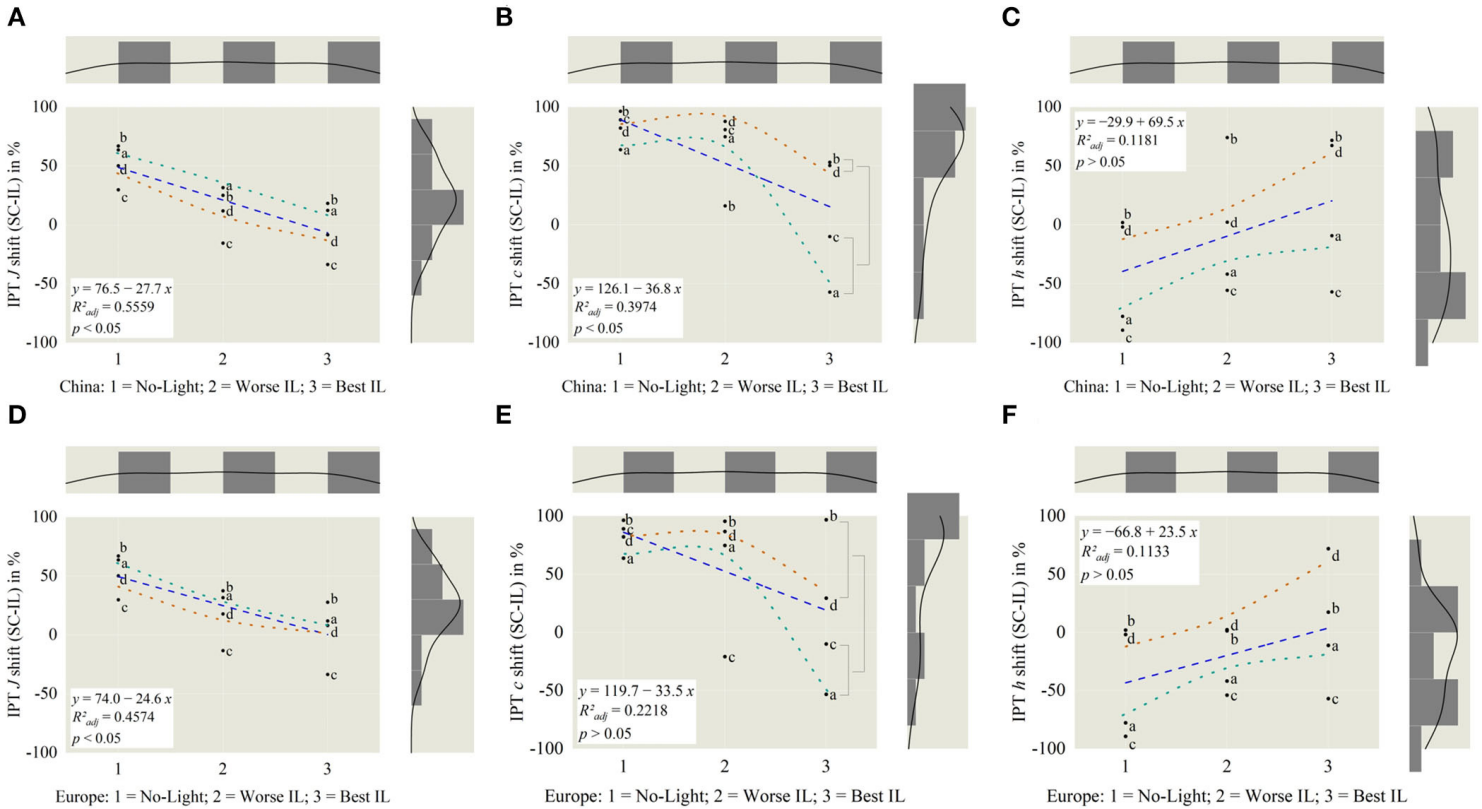
Based on *China* in IPT, for **(A)** lightness *J* = −26.5 and **(B)** chroma *c* = −31.8, slopes are significantly different from zero, resulting in a tendency from worst to best illumination. For **(C)** hue *h* = 21.8, the slope is not different. Tendency lines are added for sun city, a-marking in green and night, d-marking in brown. For reference **(D–F)**, IPT dimensions from *Europe*.

**Figure 10:**
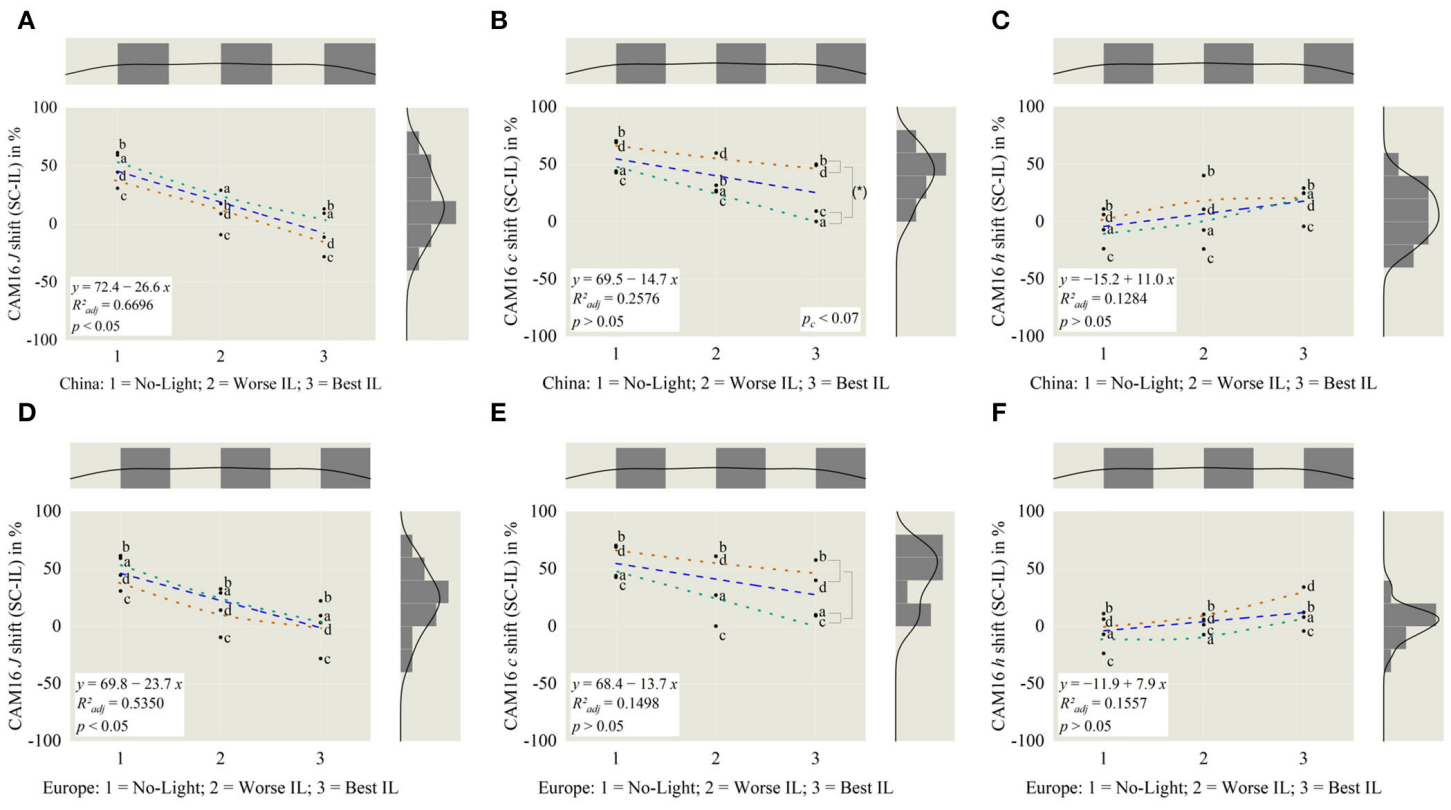
Based on *China* in CAM16, for **(A)** lightness *J* = −31.8, slope is significantly different from zero. For **(B)** chroma *c* and **(C)** hue *h*, the slopes are not different. Tendency lines like Figure 9. Chroma significantly varied (*p*_*c*_ < 0.07) between brighter and darker external scenery a and d **(B)**. For reference **(D–F)**, CAM dimensions from *Europe*.

To investigate this identified scenery relation further, regressions are calculated and modeled using polynomic functions for the IPT space, as shown in Figure 9. Here, the scenes are labeled with sun city a, countryside b, forest c and night d following the new grouping for bright–dark sceneries out of Figures 6A,D. Further, we compared investigated correlations by applying CAM16 transformations in parallel. Results for that are displayed in Figure 10.

Out of that, we conclude the following rules for in-vehicle lighting scenery:

– The brightness inside the vehicle has to be adaptive to the outside scenery, in such a way that the visual perception for outside and inside should be closed to the average of both by super sampling external light and internal originated from both natural and artificial sources, compare Figures 9A,D, 10A,D.
– Best chroma settings are achieved by adjusting in-vehicle lighting so that the external saturation is primary higher if it is outside dark and interesting, like the Shanghai night scene d, compared to a brighter interesting day-time city scene a, in which chroma should be similar for both (*p*_*c*_ < 0.07), shown in Figures 9B,E, 10B,E.
– Between outside and inside there should be no hue change (*p* > 0.05) within all different external or internal illumination settings, date and time of the day, weather, and illuminated road settings, displayed in Figures 9C,F, 10C,F.

**Figure 11:**
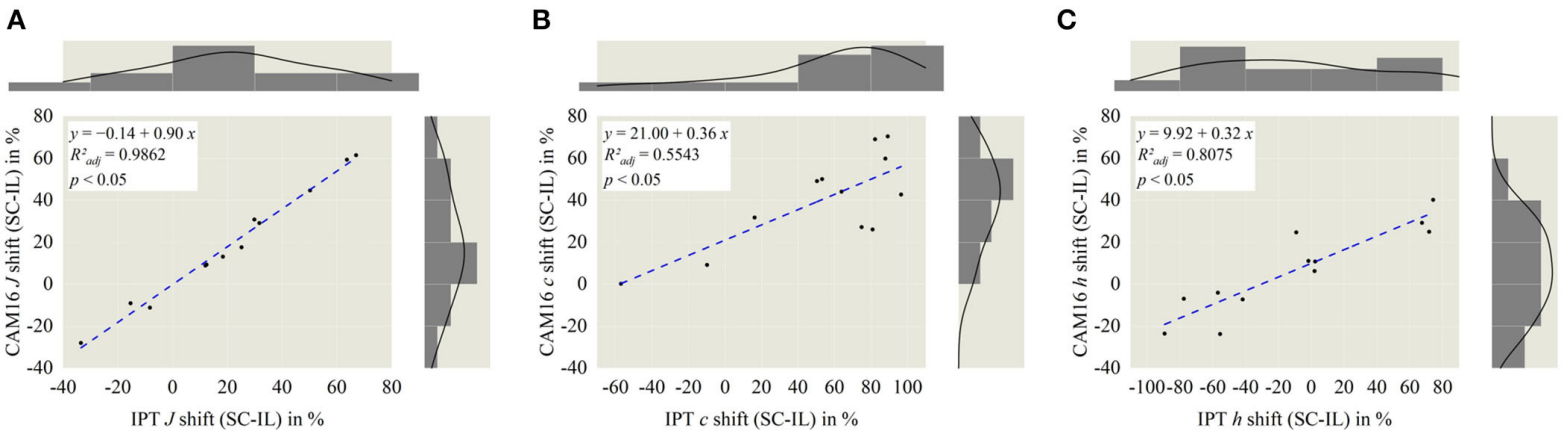
Correlation analysis between IPT and CAM16 space, separated in lightness *J* **(A)**, chroma *c* **(B)**, and hue angle *h* **(C)**. All three dimensions significantly correlate with each other (*p* < 0.05). Highest correlation is observed in *J* dimension with *R*^2^_adj_ = 0.9862.

To compare the performance of the simple IPT space compared to the enhanced color appearance model CAM16, correlations are calculated using the dataset from the larger Chinese participants and displayed in the following Figure 11.

The highest correlation with *R*^2^_*adj*_ = 0.9862 and a slope of 0.90 was investigated within the lightness dimension *J*, leading to the conclusion that for the lightness calculation with IPT and CAM16, in relative comparison, there might be fewer differences. Within the color properties of chroma *c* and hue angle *h* calculated correlation slopes are comparably small, 0.32–0.36 with *R*^2^_*adj*_ = 0.5543–0.8075, which means that absolute numbers for both dimensions are different but still transferable between both perceptional spaces.

Finally, we evaluated the last two qualitative optional questions and compared them in Section 5i−5ii. Answers to both questions are, only evaluated from the Chinese group, since there were nearly no written comments from the European group. Submitted expressions showed that our study scope was highly accepted resulting in a high wish for “surrounding-matching” or “adaptations” in other dimensions by modern in-vehicle lighting. Also, there was an ambivalence highlighted between “learn-a-lot” and “too-much” representing the participants' impression about a comprehensive study design with a valuable field of investigations.

## Discussion

Compared to our first online survey (Weirich et al., 2022) the average participating time increased by around 4 min. With around 23 min on average, the survey can be classified as comprehensive and close to the reasonable limit, which was also shown in expressions as “too-much.” On the other side, “learn-a-lot” was mentioned in the same way. Common online marketing studies can be completed in several minutes and for an optimal study time as a trade-off between high attention level and annoying, a good range of 10–15 min until 20–28 min as a maximum could be identified, which rated our study time to the nearly possible limit (Revilla and Höhne, 2020). They concluded that the survey time was primarily influenced by socio-demographics, personality, and the survey difficulty.

We were able to collect 164 full completed answers with 120 male and 44 female participants, primarily operating the survey using the Chinese language and located in China. Out of the first question part, we collected an age class difference of 1.56, meaning that the European group was on average more than 10 years older than the group of China, similar to in our first part (Weirich et al., 2022). Also, with the background of the global demographic change of our society, we kept this separation by knowing that its statistical power will be lower compared to the Chinese group based on the smaller sample size. Nearly all participants drove a vehicle before, as shown in Figure 2D with a longer driving time in Europe compared to China. To conclude, we were able to find a target group of people by spreading our online survey invitation primarily *via* social media platforms, which are self-driving, so highly connected to the vehicle field. We didn't use any commercial way to collect answers.

The first part about illumination for in-vehicle lighting was designed to answer the research question q_1_, compare the end of Section 4, about how many dimensions are needed to describe the in-vehicle illumination. Three dimensions were found by non-metrical multidimensional analysis, which is matching to the study from Flynn (Flynn et al., 1973). Similar to their study, we couldn't express a clear definition of the identified dimensions. So, we named them according to our intention as bright–dim, single–multi, and warm–cool, compared in Figure 4. Possibilities of similar attributes like a uniform–non-uniform or peripheral–overhead are other valid expressions. Besides the definition, the dissimilarity calculation based on nMDS resulted in four separated groups out of the eight different luminaire settings displayed in a 28 paired-comparison way. Since, we didn't express any kind of explanation about how the participants should judge the similarity and still create such meaningful groupings, leading to the conclusion that our three investigated dimensions are highly connected with visual attributes.

The second part applied psychological attributes, to answer research question q_2_. First, we selected three semantic differentials out of the three primary identified categories by Flynn et al. named evaluative, perceptual clarity, and spaciousness as summarized in Section Indoor illumination for user preference. In this field, we used the general evaluative impression, interest, perceptual clarity, brightness, and spaciousness we used spatial attributes, listed in Section Materials and methods. As displayed in Figure 5, mixed CCTs and mixed spot and ambient light settings achieved an outperformance in both investigated groups. Since natural daylight alone isn't able to achieve such a mixed white color and spatial light distribution, we argue here that to achieve the best psychological effect, artificial in-vehicle lighting should be also applied during day-time periods and not only as a primary orientational light during night time sessions (Wördenweber et al., 2007).

In addition, for the spatial luminaire arrangement between cooler, L4, and warmer light, L3, a higher brightness association was perceived with warmer spatial light, applying the same intensity settings for both in the pre-rendered images. For spot luminaire arrangements, brightness association was similar within all CCTs with 3,000, 4,500, and 6,000 K. In addition, L4 was significantly closer associated with L8, with no light condition resulting from the nMDS, compared in Figure 3. This result is in a first way contradictory to the common understanding of lower and higher CCTs to brightness perception. As investigated in previous studies, lower CCTs were associated primarily with brighter brightness perception (Harrington, 1954; Fotios and Levermore, 1997; Ju et al., 2012). But all studies in common, they had primary a spot light distribution, meaning, luminaires positioned at the roof and shining downside only. A wall or a complete room-filling indirect light, as we applied in our study, was missing there. So, we concluded that the commonly accepted and scientific proven rule about CCT and brightness association should be evaluated again by comparing spot and spatial luminaire arrangements as a third dimension and not only by light color and intensity settings, since we know a two-dimensional arrangement to describe psychological attributes is not enough (Flynn et al., 1973).

To evaluate the effect of external driving sceneries, which were skipped in the first two parts, four different external sceneries named sun city, countryside, forest, and night were implemented. In 124 pre-rendered images, participants were able to change the intensity of the luminaire arrangements L1, L3–L8, compared in Table 2, in five steps. First, a higher brightness selection was associated with higher brightness external sceneries (*p* < 0.05), as shown in Figures 6A,C,D,F. Within the ratings of the group *China*, all luminaire settings, besides L8, resulted in a good rating. For the group *Europe*, a moderate ranking was calculated with the cold white L3 in the forest scenery, shown in Figure 6F. Other in-vehicle lighting arrangements were similarly good rated. This means that no excellent illumination setting was found, which is our target in part three of this miniseries.

To investigate the correlation between illumination preferences and external sceneries deeper and try to model these based on tristimulus correlations in accordance with the change of the outer driving scenery, in research question q_3_, we transformed the displayed sRGB images to perceptional spaces IPT and CAM16. By applying contrasts between external and internal illumination in the dimension of lightness *J*, chroma *c*, and hue angle *h*, we were able to define applied luminaire working areas for the dimension of lightness and chroma, shown in Figures 8B,C and for hue in Figures 8E,F. Compared to overall possible areas, which were created within all luminaire and brightness settings, Figures 8A,B preferred areas were centrally scaled down resulting in a slightly- or non-overlapping area. To investigate these working areas further, we compared the three perceptual dimensions in the IPT and CAM16 space for the no-light condition, L8, the best in-vehicle, and worst in-vehicle lighting condition according to the absolute ranking in Figures 6C,F. It was clearly found, that for the lightness dimension *J*, the external and internal brightness should be similar and not like it is common today that external sceneries are much brighter than in-vehicle lighting settings with only up to 10–100 l × for a reading light function as highest luminaire (Wördenweber et al., 2007). Second, for the chroma dimension, we found partly evidence for the commonly accepted Hunt-Effect, which describes by decreasing intensity only, colors will be perceived as less saturated (Hunt, 1977). To compensate for this effect, under low-intensity settings, enhanced chroma settings should be applied, which was the latest proven (Kawashima and Ohno, 2019). By comparing scene a in the Chinese group, the interesting bright sun city scenery, with since d, an interesting dark city location, relatively spoken, external higher chroma settings should be applied for the darker scenery to compensate for the low-intensity level, following Hunt. For the brighter outside scenery, this compensation is not necessary (*p*_*c*_ < 0.07), compare Figure 10B. We assume that both sceneries are similar interesting to observe, since both showing a high detailed interesting city scenery with several colorful visual stimuli. Also, in the preference rating, L6 and L7 were significantly higher rated (*p* < 0.05) than other luminaire settings in the Chinese group, as shown in Figure 6C. In the sun city scene, L6, ambient warm-white, was higher rated compared to L7, ambient cold-white, for the night scenery d, both with *r* = 0.177–0.189, indicating a weak effect size. Anyway, the correlation between outside and inside hue contrasts could so far not fully investigated, represented also by not significant slop differences to zero (*p* > 0.05) in Figures 9C–F,10C–F. We are aiming to evaluate them in part 3 of our miniseries. Further, these guidelines took into account, that our target group is acting as passengers, so not primary driving.

Last, we compared the correlation between the simpler IPT and the enhanced CAM16 color appearance space in each of the three dimensions, displayed in Figure 11. For lightness *J*, both model approaches can be judged as nearly equal, represented in $R_{adj}^{2}$ = 0.9862. The correlation between hue angle was identified as the second-best and chroma with $R_{adj}^{2}$ = 0.5543 as the worst. We didn't use the uniform color space CAM16-UCS, since we explicitly aimed to compare the three basic visual attributes like lightness, chroma and hue separately and not a comparison between Euclidian distances. Since hue angle *h'* = *h*, defined in CAM16-UCS, there will be also no differences in this dimension at all (Li et al., 2017). By comparing CAM16-UCS with IPT, studies also confirmed our results by evaluating data in the Munsell Color system that there was a high similarity in value, as lightness, a strong similarity in hue, and the weakest correlation in chroma (Safdar et al., 2017). Coming to modern high-definition image applications, IPT also outperformed CAM16-UCS in regards to hue linearity especially in the blueish area (Zhao and Luo, 2020), concluding that taking all aspects into account including computational effort, depending on the application, the IPT space is still useful for perceptual evaluations.

Finally, we will add some study limitations. First, from the decision to conduct an external free-access online survey, participant observation was not possible. Hence, actual perceived brightness and color values were varying between participants. We tried to compensate for this effect by a large separation in three domains of brightness, color, and spatial distribution of our applied luminaire settings. Although, we collected detailed device settings, like brand, model, and actual screen brightness settings, based on the lockdown policy applied in China, a photometric measurement of the top 10 used mobile devices was until now not possible. But we are aiming to add these extensions in part 3. Within the complete study, our sample size collected by the European group was smaller compared to the Chinese group. So, we did not perform any comparison of both groups, just presented their results individually. Latest, a comparison between the presented model study and a real object study is necessary to evaluate these new findings.

## Conclusion

In this second part of our miniseries about modern in-vehicle lighting, we performed a comprehensive online survey about applied lighting for vehicle passengers, not drivers. Out of 164 collected answers from China and Europe, we were able to investigate three primary luminaire dimensions, named warm–cool, dim–dark, and multi–single. Within our six psychological attributes, mixed CCT settings of cooler and warmer white combined with mixed spatial luminaire settings outperformed all single illumination settings. Suggesting that for the most enhanced illumination experience inside a modern vehicle, daylight has to be combined with artificial luminaires day- and night-time. No light in the vehicle performed worse under all settings. Finally, through differentiation between day, night, interesting, and monotonous external sceneries with internal lighting settings, three major guidelines were concluded based on three basic perceptional attributes lighting, chroma and hue:

– External and internal brightness levels should be on average closer or equal to each other.
– If the outside scenery is dark or dim but interesting, the external chroma is primary higher than the internal, following Hunt. If the outside scenery is brighter and more interesting, chroma should be similar for both, the internal and external scenery.
– No hue shift should be observed between outside and inside lighting.

With these three basic connections between external and internal luminaire settings, we achieved a new step on the way to illumination modeling for modern in-vehicle lighting, which can be stated as the first guideline for in-vehicle light engineers. In our next controlled laboratory study, part 3, we aim at confirming mentioned conditional findings by a combination with results out of our first and this second part by adding also an investigation of adaptational effects during night and day-time, which might be an explanation for different ratings between these two time zones, which we currently found.

## Data availability statement

The original contributions presented in the study are included in the article/Supplementary material, further inquiries can be directed to the corresponding author/s.

## Ethics statement

The studies involving human participants were reviewed and approved by School of Life Science, Fudan University (No. FE22028R). Written informed consent was obtained from the participants through a consent checklist and agreed to participate in this anonymous study by self-continuing answering with the possibility to self-quit the research at any time.

## Author contributions

CW: conceptualization, methodology, software, formal analysis, and writing—original draft preparation. CW, YL, and TK: writing—review and editing. YL and TK: supervision. All authors have read and agreed to the published version of the manuscript.

## Conflict of interest

The authors declare that the research was conducted in the absence of any commercial or financial relationships that could be construed as a potential conflict of interest.

## Publisher's note

All claims expressed in this article are solely those of the authors and do not necessarily represent those of their affiliated organizations, or those of the publisher, the editors and the reviewers. Any product that may be evaluated in this article, or claim that may be made by its manufacturer, is not guaranteed or endorsed by the publisher.
