# Supplementary material for: Evidence for human-centric in-vehicle lighting: Part 2—Modeling illumination based on color-opponents
Source: Front Neurosci. 2022 Sep 27;16:969125. doi: 10.3389/fnins.2022.969125 (PMC9552885; doi:10.3389/fnins.2022.969125)
Supplement: Supplementary file 1 [file Data_Sheet_1.PDF]

S1: Luminaire Preference Rating, China

Scenery: Sun-City

Table S1. Statistical evaluation of the sun-city scenery rating, differences of the mean values $\Delta\bar{x}$, z-score, probability value *p*, effect power *r* and quantification of it. If $p < 0.05$, it is bold marked. Participants are summed up in the heading.

Luminaire Setting	Scenery: Sun-City, China, $n = 148$														
	**L3**					**L4**					**L5**				
	**$\Delta\bar{x}$**	***z***	***p* (asym.)**	***r***	**level**	**$\Delta\bar{x}$**	***z***	***p* (asym.)**	***r***	**level**	**$\Delta\bar{x}$**	***z***	***p* (asym.)**	***r***	**level**
L1	0.182	1.976	**4.81 × 10−2**	**0.162**	**weak**	0.095	1.022	0.307	not sign.	not sign.	-0.041	-0.581	0.562	not sign.	not sign.
L3	−	−	−	−	−	-0.088	-0.955	0.343	not sign.	not sign.	-0.223	-2.601	**9.30 × 10−3**	**0.214**	**weak**
L4	−	−	−	−	−	−	−	−	−	−	-0.135	-1.943	**5.18 × 10−2**	**0.160**	**weak**
L5	−	−	−	−	−	−	−	−	−	−	−	−	−	−	−
L6	−	−	−	−	−	−	−	−	−	−	−	−	−	−	−
L7	−	−	−	−	−	−	−	−	−	−	−	−	−	−	−
L8	−	−	−	−	−	−	−	−	−	−	−	−	−	−	−

Luminaire Setting	**L6**					**L7**					**L8**				
	**$\Delta\bar{x}$**	***z***	***p* (asym.)**	***r***	**level**	**$\Delta\bar{x}$**	***z***	***p* (asym.)**	***r***	**level**	**$\Delta\bar{x}$**	***z***	***p* (asym.)**	***r***	**level**
L1	-0.318	-2.966	**3.02 × 10−3**	**0.244**	**weak**	-0.142	-1.273	0.203	not sign.	not sign.	1.236	7.812	**5.55 × 10−15**	**0.642**	**strong**
L3	-0.500	-4.853	**1.22 × 10−6**	**0.399**	**medium**	-0.324	-3.000	**2.70 × 10−3**	**0.247**	**weak**	1.054	7.092	**1.32 × 10−12**	**0.583**	**strong**
L4	-0.412	-4.280	**9.37 × 10−6**	**0.352**	**medium**	-0.236	-2.478	**1.32 × 10−2**	**0.204**	**weak**	1.142	7.168	**7.62 × 10−13**	**0.589**	**strong**
L5	-0.277	-3.104	**1.65 × 10−3**	**0.255**	**medium**	-0.101	-0.846	0.398	not sign.	not sign.	1.277	7.457	**8.84 × 10−14**	**0.613**	**strong**
L6	−	−	−	−	−	0.176	2.165	**3.04 × 10−2**	**0.178**	**weak**	1.554	8.352	**0.00**	**0.687**	**strong**
L7	−	−	−	−	−	−	−	−	−	−	1.378	8.053	**8.88 × 10−16**	**0.662**	**strong**
L8	−	−	−	−	−	−	−	−	−	−	−	−	−	−	−

Scenery: Country

Table S2. Statistical evaluation of the country scenery rating, differences of the mean values $\Delta\bar{x}$, z-score, probability value *p,* effect power *r* and quantification of it. If $p < 0.05$, it is bold marked. Participants are summed up in the heading.

**Luminaire Setting**	**Scenery: Country, China, *n* = 148**														
	**L3**					**L4**					**L5**				
	$\Delta\bar{x}$	*z*	***p* (asym.)**	***r***	**level**	$\Delta\bar{x}$	*z*	***p* (asym.)**	***r***	**level**	$\Delta\bar{x}$	*z*	***p* (asym.)**	***r***	**level**
L1	-0.054	-0.666	0.515	not sign.	not sign.	0.027	0.285	0.775	not sign.	not sign.	-0.264	-2.431	**1.51 × 10−2**	**0.200**	**weak**
L3	−	−	−	−	−	0.081	0.810	0.424	not sign.	not sign.	-0.209	-2.562	**1.04 × 10−2**	**0.211**	**weak**
L4	−	−	−	−	−	−	−	−	−	−	-0.291	-3.390	**5.24 × 10−4**	**0.279**	**medium**
L5	−	−	−	−	−	−	−	−	−	−	−	−	−	−	−
L6	−	−	−	−	−	−	−	−	−	−	−	−	−	−	−
L7	−	−	−	−	−	−	−	−	−	−	−	−	−	−	−
L8	−	−	−	−	−	−	−	−	−	−	−	−	−	−	−
	**L6**					**L7**					**L8**				
	$\Delta\bar{x}$	*z*	***p* (asym.)**	***r***	**level**	$\Delta\bar{x}$	*z*	***p* (asym.)**	***r***	**level**	$\Delta\bar{x}$	*z*	***p* (asym.)**	***r***	**level**
L1	-0.345	-3.545	**2.89 × 10−4**	**0.291**	**medium**	-0.392	-4.108	**3.99 × 10−5**	**0.338**	**medium**	0.797	6.377	**1.81 × 10−10**	**0.524**	**strong**
L3	-0.291	-3.041	**2.10 × 10−3**	**0.250**	**medium**	-0.338	-3.514	**3.29 × 10−4**	**0.289**	**medium**	0.851	6.199	**5.68 × 10−10**	**0.510**	**strong**
L4	-0.372	-4.343	**5.92 × 10−6**	**0.357**	**medium**	-0.419	-4.535	**2.47 × 10−6**	**0.373**	**medium**	0.770	5.806	**6.40 × 10−9**	**0.477**	**strong**
L5	-0.081	-0.666	0.509	not sign.	not sign.	-0.128	-1.251	0.212	not sign.	not sign.	1.061	6.909	**4.89 × 10−12**	**0.568**	**strong**
L6	−	−	−	−	−	-0.047	-0.705	0.481	not sign.	not sign.	1.142	7.665	**1.78 × 10−14**	**0.630**	**strong**
L7	−	−	−	−	−	−	−	−	−	−	1.189	7.587	**3.29 × 10−14**	**0.624**	**strong**
L8	−	−	−	−	−	−	−	−	−	−	−	−	−	−	−

Scenery: Forest

Table S3. Statistical evaluation of the forest scenery rating, differences of the mean values $\Delta\bar{x}$, z-score, probability value *p,* effect power *r* and quantification of it. If $p < 0.05$, it is bold marked. Participants are summed up in the heading.

**Luminaire Setting**	**Scenery: Forest, China, *n* = 148**														
	**L3**					**L4**					**L5**				
	**$\Delta\bar{x}$**	***z***	***p* (asym.)**	***r***	**level**	**$\Delta\bar{x}$**	***z***	***p* (asym.)**	***r***	**level**	**$\Delta\bar{x}$**	***z***	***p* (asym.)**	***r***	**level**
L1	0.331	3.637	**1.97 × 10−4**	**0.299**	**medium**	0.257	3.139	**1.69 × 10−3**	**0.258**	**medium**	0.182	2.016	**4.38 × 10−2**	**0.166**	**weak**
L3	–	–	–	–	–	-0.074	-0.818	0.414	not sign.	not sign.	-0.149	-1.735	**8.23 × 10−2**	**0.143**	**weak**
L4	–	–	–	–	–	–	–	–	–	–	-0.074	-0.796	0.430	not sign.	not sign.
L5	–	–	–	–	–	–	–	–	–	–	–	–	–	–	–
L6	–	–	–	–	–	–	–	–	–	–	–	–	–	–	–
L7	–	–	–	–	–	–	–	–	–	–	–	–	–	–	–
L8	–	–	–	–	–	–	–	–	–	–	–	–	–	–	–
	**L6**					**L7**					**L8**				
	**$\Delta\bar{x}$**	***z***	***p* (asym.)**	***r***	**level**	**$\Delta\bar{x}$**	***z***	***p* (asym.)**	***r***	**level**	**$\Delta\bar{x}$**	***z***	***p* (asym.)**	***r***	**level**
L1	-0.054	-0.679	0.497	not sign.	not sign.	-0.189	-2.150	**3.17 × 10−2**	**0.177**	**weak**	0.892	6.167	**6.97 × 10−10**	**0.507**	**strong**
L3	-0.385	-3.893	**9.88 × 10−5**	**0.320**	**medium**	-0.520	-4.897	**9.72 × 10−7**	**0.403**	**strong**	0.561	4.568	**4.92 × 10−6**	**0.375**	**medium**
L4	-0.311	-3.494	**4.76 × 10−4**	**0.287**	**medium**	-0.446	-4.500	**6.80 × 10−6**	**0.370**	**medium**	0.635	4.531	**5.88 × 10−6**	**0.372**	**medium**
L5	-0.236	-2.686	**7.23 × 10−3**	**0.221**	**weak**	-0.372	-3.944	**8.01 × 10−5**	**0.324**	**medium**	0.709	4.838	**1.31 × 10−6**	**0.398**	**medium**
L6	–	–	–	–	–	-0.135	-1.661	0.097	not sign.	not sign.	0.946	6.263	**3.77 × 10−10**	**0.515**	**strong**
L7	–	–	–	–	–	–	–	–	–	–	1.081	7.206	**5.78 × 10−13**	**0.592**	**strong**
L8	–	–	–	–	–	–	–	–	–	–	–	–	–	–	–

Scenery: Night

Table S4. Statistical evaluation of the night scenery rating, differences of the mean values $\Delta\bar{x}$, z-score, probability value *p,* effect power *r* and quantification of it. If $p < 0.05$, it is bold marked. Participants are summed up in the heading.

**Luminaire Setting**	**Scenery: Night, China, *n* = 148**														
	**L3**					**L4**					**L5**				
	**$\Delta\bar{x}$**	***z***	***p* (asym.)**	***r***	**level**	**$\Delta\bar{x}$**	***z***	***p* (asym.)**	***r***	**level**	**$\Delta\bar{x}$**	***z***	***p* (asym.)**	***r***	**level**
L1	0.128	1.759	0.079	not sign.	not sign.	0.122	1.289	0.201	not sign.	not sign.	0.074	0.857	0.392	not sign.	not sign.
L3	–	–	–	–	–	-0.007	-0.235	0.816	not sign.	not sign.	-0.054	-0.591	0.563	not sign.	not sign.
L4	–	–	–	–	–	–	–	–	–	–	-0.047	-0.530	0.600	not sign.	not sign.
L5	–	–	–	–	–	–	–	–	–	–	–	–	–	–	–
L6	–	–	–	–	–	–	–	–	–	–	–	–	–	–	–
L7	–	–	–	–	–	–	–	–	–	–	–	–	–	–	–
L8	–	–	–	–	–	–	–	–	–	–	–	–	–	–	–
	**L6**					**L7**					**L8**				
	**$\Delta\bar{x}$**	***z***	***p* (asym.)**	***r***	**level**	**$\Delta\bar{x}$**	***z***	***p* (asym.)**	***r***	**level**	**$\Delta\bar{x}$**	***z***	***p* (asym.)**	***r***	**level**
L1	0.034	0.172	0.865	not sign.	not sign.	-0.128	-1.681	0.093	not sign.	not sign.	0.682	4.866	**1.14 × 10−6**	**0.400**	**strong**
L3	-0.095	-1.144	0.257	not sign.	not sign.	-0.257	-2.876	**3.70 × 10−3**	**0.236**	**weak**	0.554	4.255	**2.09 × 10−5**	**0.350**	**medium**
L4	-0.088	-0.852	0.396	not sign.	not sign.	-0.250	-2.654	**7.56 × 10−3**	**0.218**	**weak**	0.561	4.129	**3.65 × 10−5**	**0.339**	**medium**
L5	-0.041	-0.518	0.607	not sign.	not sign.	-0.203	-2.210	**2.64 × 10−2**	**0.182**	**weak**	0.608	4.551	**5.34 × 10−6**	**0.374**	**medium**
L6	–	–	–	–	–	-0.162	-2.431	**1.51 × 10−2**	**0.200**	**weak**	0.649	4.611	**4.01 × 10−6**	**0.379**	**strong**
L7	–	–	–	–	–	–	–	–	–	–	0.811	5.735	**9.75 × 10−9**	**0.471**	**strong**
L8	–	–	–	–	–	–	–	–	–	–	–	–	–	–	–

Luminaire baseline setting, L8: Comparison within Sceneries.

Table S5. Statistical evaluation of the L8 rating, differences of the mean values $\Delta\bar{x}$, z-score, probability value *p,* effect power *r* and quantification of it. If $p < 0.05$, it is bold marked. Participants are summed up in the heading.

**Sceneries**	**Luminaire setting L8, China, *n* = 148**														
	**Country**					**Forest**					**Night**				
	**$\Delta\bar{x}$**	***z***	***p* (asym.)**	***r***	**level**	**$\Delta\bar{x}$**	***z***	***p* (asym.)**	***r***	**level**	**$\Delta\bar{x}$**	***z***	***p* (asym.)**	***r***	**level**
Sun City	-0.372	-3.570	**3.57 × 10−4**	**0.293**	**medium**	-0.426	-3.859	**1.14 × 10−4**	**0.317**	**medium**	-0.838	-6.508	**7.61 × 10−11**	**0.535**	**strong**
Country	–	–	–	–	–	-0.054	-0.538	0.590	not sign.	not sign.	-0.466	-4.307	**1.65 × 10−5**	**0.354**	**medium**
Forest	–	–	–	–	–	–	–	–	–	–	-0.412	-3.365	**7.64 × 10−4**	**0.277**	**medium**
Night	–	–	–	–	–	–	–	–	–	–	–	–	–	–	–

S2: Luminaire Preference Rating, Europe

Scenery: Sun-City

Table S6. Statistical evaluation of the sun-city scenery rating, differences of the mean values $\Delta\bar{x}$, z-score, probability value *p,* effect power *r* and quantification of it. If $p < 0.08$, it is bold marked. Participants are summed up in the heading.

**Scenery: Sun-City, Europe, *n* = 16**															
**Luminaire Setting**	**L3**					**L4**					**L5**				
	**$\Delta\bar{x}$**	***z***	***p* (asym.)**	***r***	**level**	**$\Delta\bar{x}$**	***z***	***p* (asym.)**	***r***	**level**	**$\Delta\bar{x}$**	***z***	***p* (asym.)**	***r***	**level**
L1	0.250	0.930	0.325	not sign.	not sign.	0.000	0.000	1.000	not sign.	not sign.	-0.125	-0.239	0.797	not sign.	not sign.
L3	–	–	–	–	–	-0.250	-0.837	0.489	not sign.	not sign.	-0.375	-0.635	0.560	not sign.	not sign.
L4	–	–	–	–	–	–	–	–	–	–	-0.125	0.000	1.000	not sign.	not sign.
L5	–	–	–	–	–	–	–	–	–	–	–	–	–	–	–
L6	–	–	–	–	–	–	–	–	–	–	–	–	–	–	–
L7	–	–	–	–	–	–	–	–	–	–	–	–	–	–	–
L8	–	–	–	–	–	–	–	–	–	–	–	–	–	–	–

	**L6**					**L7**					**L8**				
	**$\Delta\bar{x}$**	***z***	***p* (asym.)**	***r***	**level**	**$\Delta\bar{x}$**	***z***	***p* (asym.)**	***r***	**level**	**$\Delta\bar{x}$**	***z***	***p* (asym.)**	***r***	**level**
L1	-0.250	-0.642	0.525	not sign.	not sign.	-0.438	-0.887	0.395	not sign.	not sign.	0.875	2.236	**2.25×10^{-2}**	**0.559**	**strong**
L3	-0.500	-1.029	0.336	not sign.	not sign.	-0.688	-1.653	0.125	not sign.	not sign.	0.625	1.573	0.135	not sign.	not sign.
L4	-0.250	-0.679	0.510	not sign.	not sign.	-0.438	-1.219	0.264	not sign.	not sign.	0.875	2.105	**3.20×10^{-2}**	**0.526**	**strong**
L5	-0.125	-0.486	0.795	not sign.	not sign.	-0.313	-1.061	0.363	not sign.	not sign.	1.000	1.698	**8.20×10^{-2}**	**0.424**	**strong**
L6	–	–	–	–	–	-0.188	-0.482	0.630	not sign.	not sign.	1.125	1.751	**6.84×10^{-2}**	**0.438**	**strong**
L7	–	–	–	–	–	–	–	–	–	–	1.313	2.263	**2.23×10^{-2}**	**0.566**	**strong**
L8	–	–	–	–	–	–	–	–	–	–	–	–	–	–	–

Scenery: Country

Table S7. Statistical evaluation of the country scenery rating, differences of the mean values $\Delta\bar{x}$, z-score, probability value *p*, effect power *r* and quantification of it. If $p < 0.08$, it is bold marked. Participants are summed up in the heading.

**Luminaire Setting**	**Scenery: Country, Europe, *n* = 16**														
	**L3**					**L4**					**L5**				
	$\Delta\bar{x}$	*z*	***p* (asym.)**	***r***	**level**	$\Delta\bar{x}$	*z*	***p* (asym.)**	***r***	**level**	$\Delta\bar{x}$	*z*	***p* (asym.)**	***r***	**level**
L1	-0.063	-0.366	0.797	not sign.	not sign.	-0.438	-1.219	0.264	not sign.	not sign.	-0.500	-1.113	0.271	not sign.	not sign.
L3	–	–	–	–	–	-0.375	-0.926	0.359	not sign.	not sign.	-0.438	-0.968	0.371	not sign.	not sign.
L4	–	–	–	–	–	–	–	–	–	–	-0.063	-0.298	1.000	not sign.	not sign.
L5	–	–	–	–	–	–	–	–	–	–	–	–	–	–	–
L6	–	–	–	–	–	–	–	–	–	–	–	–	–	–	–
L7	–	–	–	–	–	–	–	–	–	–	–	–	–	–	–
L8	–	–	–	–	–	–	–	–	–	–	–	–	–	–	–
	**L6**					**L7**					**L8**				
	$\Delta\bar{x}$	*z*	***p* (asym.)**	***r***	**level**	$\Delta\bar{x}$	*z*	***p* (asym.)**	***r***	**level**	$\Delta\bar{x}$	*z*	***p* (asym.)**	***r***	**level**
L1	-0.500	-1.038	0.333	not sign.	not sign.	-0.500	-1.173	0.281	not sign.	not sign.	0.688	1.596	0.115	not sign.	not sign.
L3	-0.438	-0.994	0.340	not sign.	not sign.	-0.438	-1.140	0.283	not sign.	not sign.	0.750	2.132	**3.03×10^{-2}**	**0.533**	**strong**
L4	-0.063	-0.209	0.830	not sign.	not sign.	-0.063	-0.155	0.887	not sign.	not sign.	1.125	2.340	**1.42×10^{-2}**	**0.585**	**strong**
L5	0.000	-0.209	0.830	not sign.	not sign.	0.000	-0.181	0.812	not sign.	not sign.	1.188	2.243	**1.76×10^{-2}**	**0.561**	**strong**
L6	–	–	–	–	–	0.000	0.000	1.000	not sign.	not sign.	1.188	2.455	**9.28×10^{-3}**	**0.614**	**strong**
L7	–	–	–	–	–	–	–	–	–	–	1.188	2.276	**1.53×10^{-2}**	**0.569**	**strong**
L8	–	–	–	–	–	–	–	–	–	–	–	–	–	–	–

Scenery: Forest

Table S8. Statistical evaluation of the forest scenery rating, differences of the mean values $\Delta\bar{x}$, z-score, probability value *p,* effect power *r* and quantification of it. If $p < 0.08$, it is bold marked. Participants are summed up in the heading.

**Luminaire Setting**	**Scenery: Forest, Europe, *n* = 16**														
	**L3**					**L4**					**L5**				
	**$\Delta\bar{x}$**	***z***	***p* (asym.)**	***r***	**level**	**$\Delta\bar{x}$**	***z***	***p* (asym.)**	***r***	**level**	**$\Delta\bar{x}$**	***z***	***p* (asym.)**	***r***	**level**
L1	0.563	1.726	**8.98 × 10−2**	**0.432**	**strong**	0.313	0.582	0.595	not sign.	not sign.	0.063	0.120	0.892	not sign.	not sign.
L3	–	–	–	–	–	-0.250	-0.882	0.484	not sign.	not sign.	-0.500	-1.242	0.239	not sign.	not sign.
L4	–	–	–	–	–	–	–	–	–	–	-0.250	-0.605	0.574	not sign.	not sign.
L5	–	–	–	–	–	–	–	–	–	–	–	–	–	–	–
L6	–	–	–	–	–	–	–	–	–	–	–	–	–	–	–
L7	–	–	–	–	–	–	–	–	–	–	–	–	–	–	–
L8	–	–	–	–	–	–	–	–	–	–	–	–	–	–	–
	**L6**					**L7**					**L8**				
	**$\Delta\bar{x}$**	***z***	***p* (asym.)**	***r***	**level**	**$\Delta\bar{x}$**	***z***	***p* (asym.)**	***r***	**level**	**$\Delta\bar{x}$**	***z***	***p* (asym.)**	***r***	**level**
L1	-0.188	-0.098	0.909	not sign.	not sign.	-0.250	-0.455	0.700	not sign.	not sign.	0.750	1.932	**6.15 × 10−2**	**0.483**	**strong**
L3	-0.750	-2.029	**4.74 × 10−2**	**0.507**	**strong**	-0.813	-2.161	**2.73 × 10−2**	**0.540**	**strong**	0.188	0.611	0.553	not sign.	not sign.
L4	-0.500	-1.137	0.266	not sign.	not sign.	-0.563	-1.445	0.170	not sign.	not sign.	0.438	1.394	0.151	not sign.	not sign.
L5	-0.250	-0.770	0.468	not sign.	not sign.	-0.313	-0.785	0.459	not sign.	not sign.	0.688	1.342	0.185	not sign.	not sign.
L6	–	–	–	–	–	-0.063	-0.283	0.777	not sign.	not sign.	0.938	1.826	**7.57 × 10−2**	**0.457**	**strong**
L7	–	–	–	–	–	–	–	–	–	–	1.000	2.022	**4.79 × 10−2**	**0.506**	**strong**
L8	–	–	–	–	–	–	–	–	–	–	–	–	–	–	–

Scenery: Night

Table S9. Statistical evaluation of the night scenery rating, differences of the mean values $\Delta\bar{x}$, z-score, probability value *p,* effect power *r* and quantification of it. If $p < 0.08$, it is bold marked. Participants are summed up in the heading.

Luminaire Setting	**Scenery: Night, Europe, *n* = 16**														
	**L3**					**L4**					**L5**				
	**$\Delta\bar{x}$**	***z***	***p* (asym.)**	***r***	**level**	**$\Delta\bar{x}$**	***z***	***p* (asym.)**	***r***	**level**	**$\Delta\bar{x}$**	***z***	***p* (asym.)**	***r***	**level**
L1	0.188	0.749	0.449	not sign.	not sign.	0.313	1.035	0.371	not sign.	not sign.	0.250	0.744	0.531	not sign.	not sign.
L3	–	–	–	–	–	0.125	0.212	0.848	not sign.	not sign.	0.063	0.000	1.000	not sign.	not sign.
L4	–	–	–	–	–	–	–	–	–	–	-0.063	-0.189	1.000	not sign.	not sign.
L5	–	–	–	–	–	–	–	–	–	–	–	–	–	–	–
L6	–	–	–	–	–	–	–	–	–	–	–	–	–	–	–
L7	–	–	–	–	–	–	–	–	–	–	–	–	–	–	–
L8	–	–	–	–	–	–	–	–	–	–	–	–	–	–	–
	**L6**					**L7**					**L8**				
	**$\Delta\bar{x}$**	***z***	***p* (asym.)**	***r***	**level**	**$\Delta\bar{x}$**	***z***	***p* (asym.)**	***r***	**level**	**$\Delta\bar{x}$**	***z***	***p* (asym.)**	***r***	**level**
L1	0.063	0.052	0.910	not sign.	not sign.	-0.125	-0.624	0.617	not sign.	not sign.	0.438	0.982	0.322	not sign.	not sign.
L3	-0.125	-0.300	0.832	not sign.	not sign.	-0.313	-0.960	0.348	not sign.	not sign.	0.250	0.405	0.700	not sign.	not sign.
L4	-0.250	-0.789	0.516	not sign.	not sign.	-0.438	-1.269	0.195	not sign.	not sign.	0.125	0.122	0.824	not sign.	not sign.
L5	-0.188	-0.864	0.465	not sign.	not sign.	-0.375	-1.066	0.281	not sign.	not sign.	0.188	0.250	0.836	not sign.	not sign.
L6	–	–	–	–	–	-0.188	-0.275	0.784	not sign.	not sign.	0.375	0.852	0.438	not sign.	not sign.
L7	–	–	–	–	–	–	–	–	–	–	0.563	1.458	0.201	not sign.	not sign.
L8	–	–	–	–	–	–	–	–	–	–	–	–	–	–	–

Luminaire baseline setting, L8: Comparison within Sceneries.

Table S10. Statistical evaluation of the L8 rating, differences of the mean values $\Delta\bar{x}$, z-score, probability value *p,* effect power *r* and quantification of it. If $p < 0.08$, it is bold marked. Participants are summed up in the heading.

Sceneries	**Luminaire setting L8, Europe, *n* = 16**														
	**Country**					**Forest**					**Night**				
	**$\Delta\bar{x}$**	***z***	***p* (asym.)**	***r***	**level**	**$\Delta\bar{x}$**	***z***	***p* (asym.)**	***r***	**level**	**$\Delta\bar{x}$**	***z***	***p* (asym.)**	***r***	**level**
Sun City	-0.125	-0.270	0.787	not sign.	not sign.	-0.125	-0.323	0.774	not sign.	not sign.	-1.000	-1.752	**7.81×10^{-2}**	**0.438**	**strong**
Country	–	–	–	–	–	0.000	-0.142	1.000	not sign.	not sign.	-0.875	-2.319	**2.15×10^{-2}**	**0.580**	**strong**
Forest	–	–	–	–	–	–	–	–	–	–	-0.875	-2.101	**4.61×10^{-2}**	**0.525**	**strong**
Night	–	–	–	–	–	–	–	–	–	–	–	–	–	–	–
